# Tumor-associated macrophages induced spheroid formation by CCL18-ZEB1-M-CSF feedback loop to promote transcoelomic metastasis of ovarian cancer

**DOI:** 10.1136/jitc-2021-003973

**Published:** 2021-12-25

**Authors:** Lingli Long, Yue Hu, Tengfei Long, Xiaofang Lu, Ying Tuo, Yubing Li, Min Wang, Zunfu Ke

**Affiliations:** 1 Translation Medicine Center, Sun Yat-sen University First Affiliated Hospital, Guangzhou, China; 2 Department of Gynaecology and Obstetrics, Sun Yat-Sen Memorial Hospital, Guangzhou, China; 3 Department of Pathology, The Seventh Affiliated Hospital Sun Yat-sen University, Shenzhen, China; 4 Department of Pathology, Sun Yat-sen University First Affiliated Hospital, Guangzhou, China; 5 The Reproductive Center, Sun Yat-sen University First Affiliated Hospital, Guangzhou, China; 6 Interdepartmental Program in Vascular Biology and Therapeutics, Department of Pathology, Yale University School of Medicine, New Haven, Connecticut, USA; 7 Molecular Diagnosis and Gene Testing Center, The First Affiliated Hospital, Sun Yat-Sen University, Guangzhou, China

**Keywords:** macrophages, tumor microenvironment

## Abstract

**Background:**

Ovarian cancer (OvCa)-tumor-associated macrophages (TAMs) spheroids are abundantly present within ascites of high malignant patients. This study investigated the mutual interaction of OvCa cells and TAMs in the spheroids.

**Methods:**

Three-dimensional coculture system and transwell coculture system were created to mimic the OvCa and TAMs in spheroids and in disassociated state. Transwell-migration assay and scratch wound healing assay were used to measure the invasive and migratory capacity. Western blot, quantitative reverse transcription-PCR and immunostaining were used to measure the mesenchymal and epithelial markers. Flow cytometry was used to assess the polarization of TAMs. Also, the differential gene expression profile of OvCa cells and OvCa cells from spheroids were tested by RNA-sequence. Finally, the ovarian mice models were constructed by intraperitoneal injection of ID8 or OvCa-TAMs spheroids.

**Results:**

Our results indicated that the formation of OvCa-TAMs spheroids was positive related to the malignancy of OvCa cells. M2-TAMs induced the epithelial-mesenchymal transition of OvCa cells by releasing chemokine (C-C motif) ligand 18 (CCL18) in the spheroids. While, CCL18 induced macrophage colony-stimulating factor (M-CSF) transcription in OvCa cells through zinc finger E-box-binding homeobox 1 (ZEB1). This study further indicated that M-CSF secreted by OvCa cells drived the polarization of M2-TAMs. Therefore, a CCL18-ZEB1-M-CSF interacting loop between OvCa cells and TAMs in the spheroids was identified. Moreover, with blocking the expression of ZEB1 in the OvCa cell, the formation of OvCa-TAMs spheroids was impeded. In the ovarian mice models, the formation of OvCa-TAMs spheroids in the ascites was promoted by overexpressing of ZEB1 in OvCa cells, which resulted in faster and earlier transcoelomic metastasis.

**Conclusion:**

These findings suggested that the formation of OvCa-TAMs spheroids resulted in aggressive phenotype of OvCa cells, as a specific feedback loop CCL18-ZEB1-M-CSF in it. Inhibition of ZEB1 reduced OvCa-TAMs spheroids in the ascites, impeding the transcoelomic metastasis and improving the outcome of ovarian patients.

## Introduction

Ovarian cancers (OvCa) have the highest lethal rate among gynecological malignancies, because most of them are diagnosed after metastasized.^1^ The 5-year survival rates for stage III and IV OvCa are less than 30%.^2^ Although cytoreductive surgery and subsequent paclitaxel/carboplatin chemotherapy have high initial response rate of OvCa patients, the overall survival rate has not been extended because of the drug resistant disease and high relapse rate. Beside lymphatic or hematogeneous route, transcoelomic metastasis, over 70% of patients with diffuse multifocal intraperitoneal metastasis and malignant ascites, have been considered as a significant reason of the greatest recurrence and mortality of OvCa.^3^ Thus, it is important to develop novel therapeutic target of OvCa transcoelomic metastasis by studying the cellular and molecular mechanism of ovarian tumor metastasis in the ascites.

Ascites consist of different kinds cells, such as, infiltrating ovarian tumor cells and immune cells which release a large number of cytokines and extracellular vesicles to constitute a cross-linking dialog network in the tumor metastasis microenvironment.^4^ Tumor-associated macrophages (TAMs) have been identified as the major population of immune cells in the ascites environment, which have highly plastic characters, with two main subtypes: M1-like anti-tumorigenesis and M2-like protumorigenesis.^5^ M2-TAMs generally express the markers: mannose receptor (MR, CD206) and scavenger receptor class B (CD163), and release immunosuppressive factors. Previous studies showed that M2-TAMs in the ascites were strongly and positively association with OvCa transcoelomic metastasis.^6 7^ Indeed, M2-like TAMs play their protumoral functions by releasing cytokines, enzymes and chemokines, to directly improve OvCa spheroid formation and adherence of cancer cells to the metastatic sites. When ovarian tumor cells detach the primary site and enter the ascites, they can form spheroids to escape the killing effect of immune cells and get the support of growth.^8^ Therefore, spheroids’s formation is inseparable from the role of OvCa transcoelomic metastasis. A further study found that in the OvCa spheroids, a large number of TAMs located at the center and adhered with the surrounding tumors promote OvCa cell proliferation by producing growth factors.^9^ Previous study showed that TAMs were always infiltrated at the invasive front of advanced tumors, where epithelial-mesenchymal transition (EMT) of cancer cells always appeared.^10^ After undergoing the process of EMT, the epithelial features of carcinoma cells will be lost, while stem cell-like features and invasive characters will be acquired.^11^ Accordingly, we suspect TAMs in the center of OvCa spheroids enhance the malignance of surrounding tumor cells by triggering EMT process. Nevertheless, interpretation of the significance of the OvCa-TAMs spheroids for EMT of OvCa remains unclear.

Previous study indicated that EMT-inducing transcription factors (EMT-TFs) zinc finger E-box-binding homeobox 1 (ZEB1), which was highly expressed in invasive cells at the front of carcinomas, are critical inducer of EMT in cancer cells. ZEB1 activated or inhibited gene expression via targeting to the regulatory regions of its down-stream genes. Other study showed that ZEB1 as a critical regulator promoted TAMs’ protumoral functions.^12 13^ Moreover, Chemokine (C-C motif) ligand 18 (CCL18), a chemokine predominantly secreted by M2-TAMs, is related to the metastasis and poor prognosis in breast cancer.^14 15^ Our results found that in OvCa-TAMs spheroids, CCL18 released by TAMs directly induced the expression of ZEB1 in OvCa.

Our in vivo and in vitro molecular studies indicated that these spheroids forming OvCa cells underwent EMT, which promoted OvCa transcoelomic metastasis. Importantly, in the TAMs-OvCa spheroids, ZEB1 not only improved the process of EMT, but also was demanded for sustaining macrophage colony-stimulating factor (M-CSF) to stimulate the polarization of M2-TAMs. The unique interaction loop (CCL18-ZEB1-M-CSF) between TAMs and OvCa was found in the spheroids. Targeting ZEB1 decreased OvCa malignance by impairing the formation of TAMs-OvCa spheroids. These results indicated a profound role of ZEB1 in driving OvCa-TAMs crosstalk and facilitating the formation of TAMs-OvCa spheroids. Clinically, ZEB1 can be considered as a molecular target for OvCa therapy.

## Materials and method

The complete experimental protocols are described in online supplemental material.

10.1136/jitc-2021-003973.supp1 Supplementary data

## Result

### OvCa-TAMs spheroids are positively associated with the malignancy of OvCa

In our study, low malignancy OvCa patient was defined as only abdominal cavity metastasis, and the expressions of Ki67 and p53 in the tumor were less than 20%. Otherwise, patients with more than one metastatic site, and higher than 20% expressions of Ki67 and p53 in the tumor were defined as high malignancy (online supplemental table 1). Our study first found that macrophages were recruited around the cancer cells at the front of the ovarian solid tumors, in which the rate of macrophages and tumor cells were higher in high malignancy OvCa patients than that in low malignancy OvCa patients (online supplemental figure 1A, B). These findings implied an interesting speculation that the recruitment of macrophages was related to the metastatic process of ovarian tumors. In order to prove this hypothesis, macrophages in the ascitic fluid were further analyzed. The result showed that many macrophages were recruited in the ascitic fluid, and most of them were M2-TAMs (CD206^+^) in high malignancy patients (online supplemental figure 1C-E). Tumor spheroids were also observed in the ascitic fluid of OvCa patients (online supplemental figure 1F). There were more spheroids with bigger size in the high malignancy patients. Macrophages located at the center of the spheroids were only observed in the high malignancy patients (online supplemental figure 1G, H). It is reasonable to suspect that the OvCa-TAMs spheroids were related to the malignancy of OvCa, but the details have still unknown.

### The interaction of TAMs and OvCa cells in spheroids is stronger than them in dissociated state

**Figure 1:**
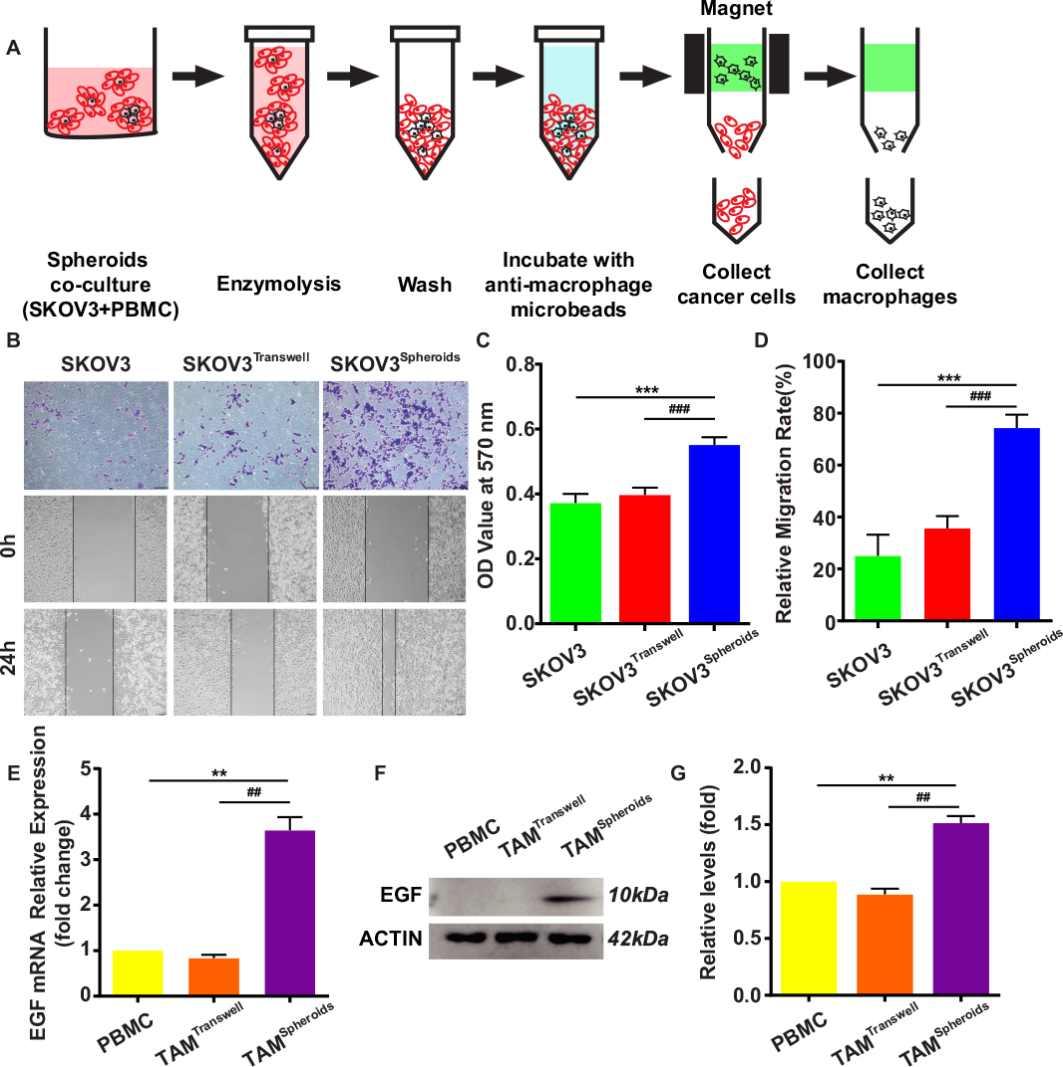
SKOV3 from OvCa-TAMs spheroids show more aggressive pattern. (A) Schematic representation depicting the separation between peripheral blood monocyte cells (PBMC; black) and ovarian cancer cells (red) from OvCa-TAMs spheroids in 3D coculture by magnetic micro-beads isolation kits (MACS). (B) The invasive and migratory capacity of SKOV3 from the OvCa-TAMs spheroid or transwell were measured. (C) The quantitation of transwell-migration assay for SKOV3 from the OvCa-TAMs spheroid or transwell (n=4 biologically independent samples per group and an average of five fields acquired from each sample). (D) The quantitation of scratch wound healing assay (n=4 biologically independent samples per group and an average of five fields acquired from each sample). (E–G) PBMC were cocultured with OvCa cells in spheroid or in transwell for 12 hours. (E) Gene expression of EGF in TAMs was determined by qRT-PCR. PBMC were used as controls. Relative gene expression is presented as fold change in relation to monocytes as 1.0. n=3. (F) Protein level of EGF were determined by Western blot. (G) Relative protein expression is presented as fold change in relation to PBMC as 1.0. n=3. Data are presented as means±SD. **P < 0.01 and ***p < 0.001 against control group, ##p < 0.01 and ###p < 0.001 against TAM in the transwell group.(two-sided Student’s t-test). 3D, three-dimensional; EGF, epidermal growth factor; OvCa, ovarian cancer; qRT-PCR, quantitative reverse transcription-PCR; TAMs, tumor-associated macrophages.

The formation of OvCa spheroid has been proven as an important step of transcoelomic metastasis.^3 16^ Previous study indicated TAMs located at the center of all the spheroids by measuring cell components in spheroids isolated from ascites of 128 cases of stage III OvCa patients.^9^ To mimic OvCa cells and TAMs in spheroids and in dissociated state, we established a spheroid three-dimensional (3D) coculture system and transwell co-culture system. The SKOV3 from spheroids were isolated by magnetic microbeads isolation kit (figure 1A). Comparing with SKOV3 alone, the invasive and migratory capacity of SKOV3 isolated from spheroids was significantly increased, but not from the transwell coculture system after 24 hours coculture (figure 1B–D). Moreover, epidermal growth factor (EGF) was highly expressed in TAMs from spheroid, but not in TAMs from transwell co-culture system (figure 1E–G). This data suggested that the growth of OvCa cells in the spheroids could be improved by the TAMs-secreted EGF. Therefore, TAMs induced protumoral and malignant phenotype of OvCa cells in the spheroids, but not in the dissociated state, which suggested a specific regulatory mechanism between TAMs and OvCa cells in the spheroids.

**Figure 2:**
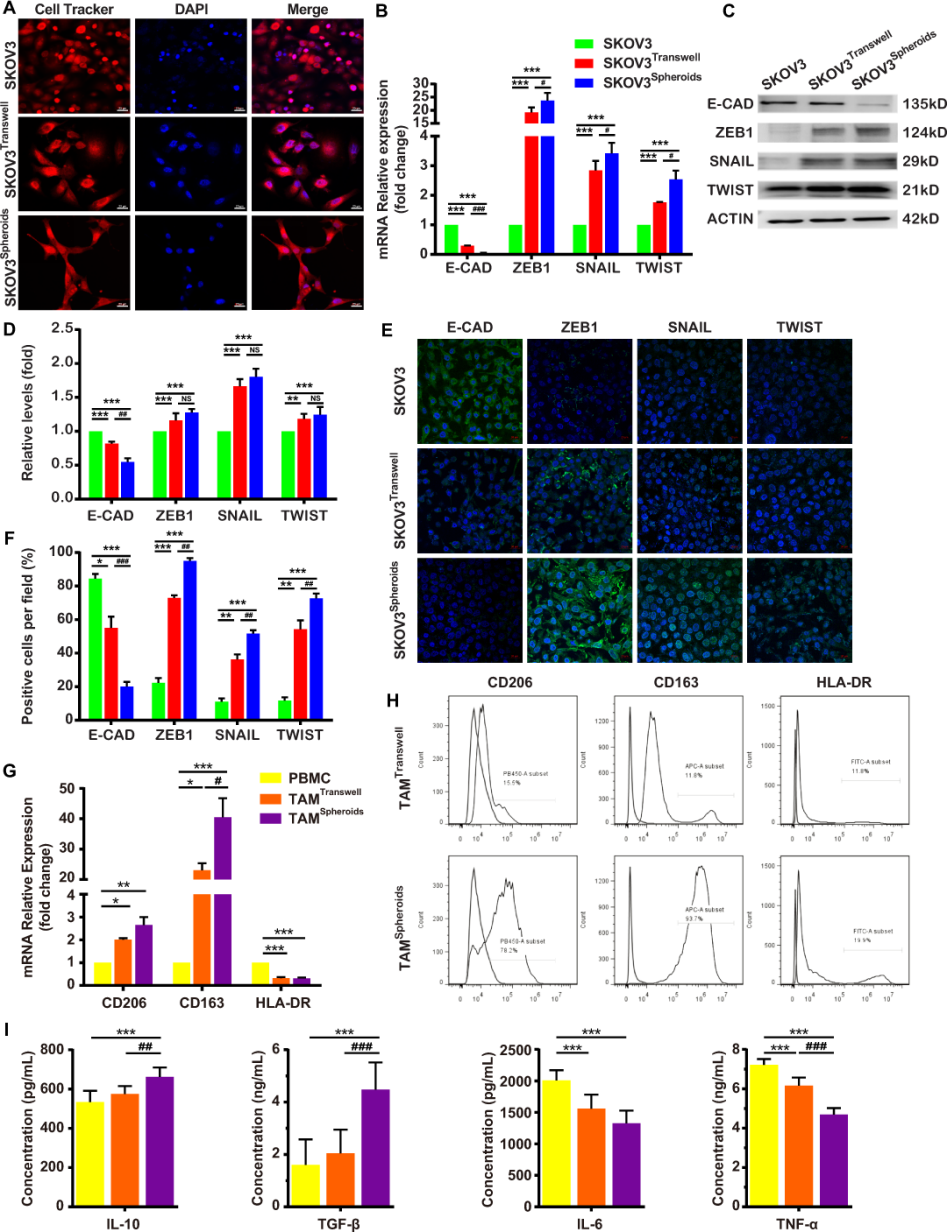
The interaction between OvCa and TAMs in the spheroids. (A–F) TAMs induce the EMT of OvCa cells in spheroids and transwell at 24 hours after coculture. (A) The morphology of OvCa cells were showed by cell tracker. (B) E-CAD, ZEB-1, SNAIL and TWIST mRNA levels in OvCa cells were detected by qRT-PCR. (C, D) The protein levels of E-CAD, ZEB-1, SNAIL and TWIST were confirmed by Western blot. (E, F) OvCa cells were subjected to immunostaining with anti-E-CAD, anti-SNAIL, anti-TWIST and DAPI, followed by confocal imaging. (G, H) Spheroidal OvCa cells induced macrophages to M2 subtype TAMs at 24 hours after coculture. (G) qRT-PCR analysis of genes involved in M2-TAM markers (CD 206 & CD 163) and a M1-TAM marker (HLA-DR) in the spheroids and transwell. (H) FCM measurement of CD206^+^ macrophages, CD163^+^ macrophages and HLA-DR^+^ macrophages in the spheroids and transwell. (I) ELISA measurement of IL-10, TGF-β, IL-6 and TNF-α in the spheroids and transwell. Data are presented as means±SD. *P<0.05; **p<0.01 and ***p < 0.001 against control group, #p < 0.5; ##p < 0.01 and ###p < 0.001 against SKOV3 in transwell group (two-sided Student’s t-test). E-CAD, E-cadherin; EMT, epithelial-mesenchymal transition; FCM, flow cytometry; IL-10, interleukin-10; OvCa, ovarian cancer; qRT-PCR, quantitative reverse transcription-PCR; TAMs, tumor-associated macrophages; TGF-β, transforming growth factor β; TNF-α, tumor necrosis factor-α.

OvCa-TAMs spheroids enhanced the early metastases, but dissociated OvCa cells in the ascites seldom showed the similar effects.^9^ The reasons of the different metastatic ability of spheroidal OvCa cells and dissociated OvCa cells have been unknown. Recently, many studies have reported that EMT plays an essential role in cancer cell metastatic dissemination events.^17^ After 24 hours coculture with TAMs in spheroids and transwell, the morphology of SKOV3 isolated from spheroids was switched into spindle, a feature of mesenchymal cells, but SKOV3 from transwell still showed epithelial morphology (figure 2A). SKOV3 in spheroids increasingly expressed the mesenchymal markers (ZEB1, SNAIL, and TWIST) and seldom expressed epithelial marker E-cadherin. On the other hand, SKOV3 from transwell expressed both the mesenchymal markers and epithelial marker. Especially, the expression of ZEB1 was dramatically increased in both co-culture systems (figure 2B–F). These evidences suggested that TAMs could induce full-EMT process of OvCa cells in spheroids, but only induced partial-EMT process of OvCa cells in transwell.

The accumulation of M2 subtype TAMs have been reported to improve the transcoelomic metastasis of OvCa.^18^ The next step, the polarization of macrophages with OvCa cells in the spheroids and transwell were compared. Figure 2G, H shows that much more CD206^+^ TAMs and CD163^+^ TAMs were induced in spheroids than in transwell (CD206: 78.2% vs 15.5%; CD163: 93.7% vs 11.8%, respectively). The TAMs in the spheroids had M2 functions were measured by the higher expression of anti-inflammatory cytokines, such as interleukin-10 (IL-10) and TGF-β, and the decreased expression of inflammatory cytokines, such as IL-6 and tumor necrosis factor-α (figure 2I). This study indicated that the formation of OvCa-TAMs spheroids was a powerful step to induce full-EMT process of OvCa cells and the polarization of M2-TAMs.

### OvCa cells produced M-CSF to polarize M2-TAMs in spheroids

To understand the molecular mechanism whereby OvCa cells and macrophages have stronger interaction in the spheroids than in the dissociated state, different cytokines secreted by macrophages and OvCa cells in spheroids and in dissociated state were compared. After 120 hours coculture, macrophages and OvCa cells were separately cultured in the serum-free medium for 12 hours. Seven cytokines were different expressed in the CM of SKOV3 alone, SKOV3^transwell^, and SKOV3^spheroids^ (online supplemental figure 2A, B), in which macrophage CSF (M-CSF) and granulocyte macrophage CSF (GM-CSF) were reported to induce M2 polarization and M1 polarization respectively.^19^ Comparing with OvCa^transwell^, higher expression of M-CSF and lower expression of GM-CSF in the OvCa^spheroids^ were confirmed by quantitative reverse transcription PCR (qRT-PCR) and ELISA (online supplemental figure 2C-F). It is reasonable to suspect that OvCa cells in the spheroids induce macrophages polarize into M2-TAMs by secreting M-CSF and inhibiting GM-CSF.

### TAMs produced CCL18 to induce EMT of OvCa cells in spheroids

Compared with TAMs^transwell^, three cytokines were increased in the TAMs^spheroid^, including CCL18, C-C motif chemokine ligand 8 (CCL8/MCP2) and beta-chemokine exodus-1 (MIP-3), in which CCL18 increased the most (online supplemental figure 2B). The higher expression of CCL18 in the TAMs^spheroid^ than in the TAMs^transwell^ was confirmed by qRT-PCR and ELISA (online supplemental figure 2G, H). Previous studies showed that CCL18 stimulates the EMT of breast cancer cells.^20^ Therefore, we suspected that macrophages in the spheroids induce the EMT of OvCa cells by releasing CCL18. All of these resluts show that OvCa cells and macrophages respectively produced higher level of M-CSF and CCL18 in the spheroids, which is possibly related to the EMT process of OvCa cells and M2 polarization of macrophages.

The chemokine receptor 8 (CCR8), a receptor of CCL18, was activated in the OvCa cells after coculture with TAMs, and had higher expression in the spheroids than in the transwell (online supplemental figure 3A-E). Another receptor of CCL18, PITPNM3, was seldom expressed in the ovarian cells, primary OvCa cells, metastatic OvCa cells and OvCa cells in the ascites (online supplemental figure 3F). Therefore, TAMs possibly produce CCL18 to activate and bind CCR8 in OvCa cells to regulate OvCa cells physiological and pathological functions.

### TAMs interact with OvCa cells and upregulation M-CSF through activation of the CCL18/ZEB1 pathway

**Figure 3:**
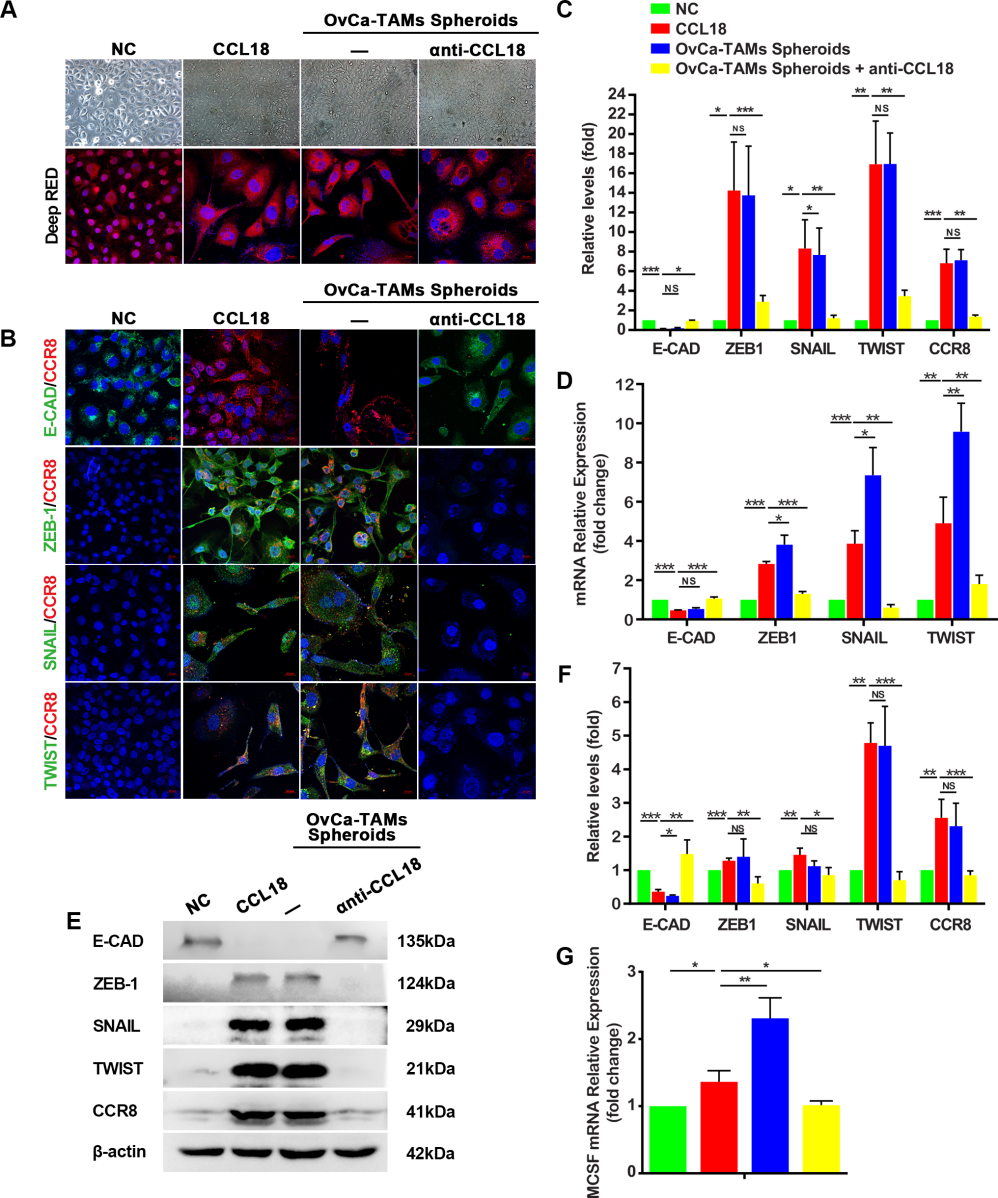
TAMs induced EMT of SKOV3 by activating CCR8. (A) The morphology of SKOV3, SKOV3 treated by CCL18, SKOV3 from OvCa-TAMs spheroids, SKOV3 from OvCa-TAMs spheroids treated by anti-CCL18. (B) Immunostaining showed the coexpression of CCR8 and EMT markers in all groups. (C) The quantification of immunostaining images. (D, E) qRT-PCR and Western bot analysis the gene and protein expression of CCR8 and EMT markers in all groups. (F) Relative protein levels of CCR8 and EMT markers were quantified. (G) The expression of M-CSF in all groups were compared by qRT-PCR. The data are presented as means±SD, n=5 independent experiments, significant difference are indicated (*p<0.05,**p < 0.01 and ***p < 0.001 against control). CCR8, Chemokine (C-C motif) ligand 18 (CCL18); E-CAD, E-cadherin; EMT, epithelial-mesenchymal transition; M-CSF, macrophage colony-stimulating factor; OvCa, ovarian cancer; qRT-PCR, quantitative reverse transcription-PCR; TAMs, tumor-associated macrophages.

In our study, SKOV3^spheroids^ or CCL18-treated SKOV3 showed mesenchymal-like changes. Their morphology turned from circular shape to spindle one, with reduction of epithelial marker, and increase of mesenchymal markers. Moreover, anti-CCL18 neutralizing antibody subdued the activation of CCR8 and the EMT process (figure 3A–F). Interestingly, the mRNA results showed that the expression of M-CSF was increased in SKOV3^spheroids^ and CCL18-treated SKOV3, but the expression was attenuated after the treatment of anti-CCL18, which suggestes that CCL18 not only induces the EMT process of OvCa cells in spheroids, but also regulates the expression of M-CSF (figure 3G).

To further confirm the regulations of CCL18 on SKOV3 through CCR8, R243, the inhibitor of CCR8, was used to treat SKOV3. The results showed that CCL18 could not induce EMT process in R243-treated SKOV3 (online supplemental figure 4A-E). Additionally, in the R243-treated SKOV3, the expression of M-CSF was not increased by adding CCL18 (online supplemental figure 4F). In summary, our data suggest a novel insight that TAMs-secreted CCL18 induces the EMT and increases the expression of M-CSF of OvCa in spheroids through activating CCR8.

**Figure 4:**
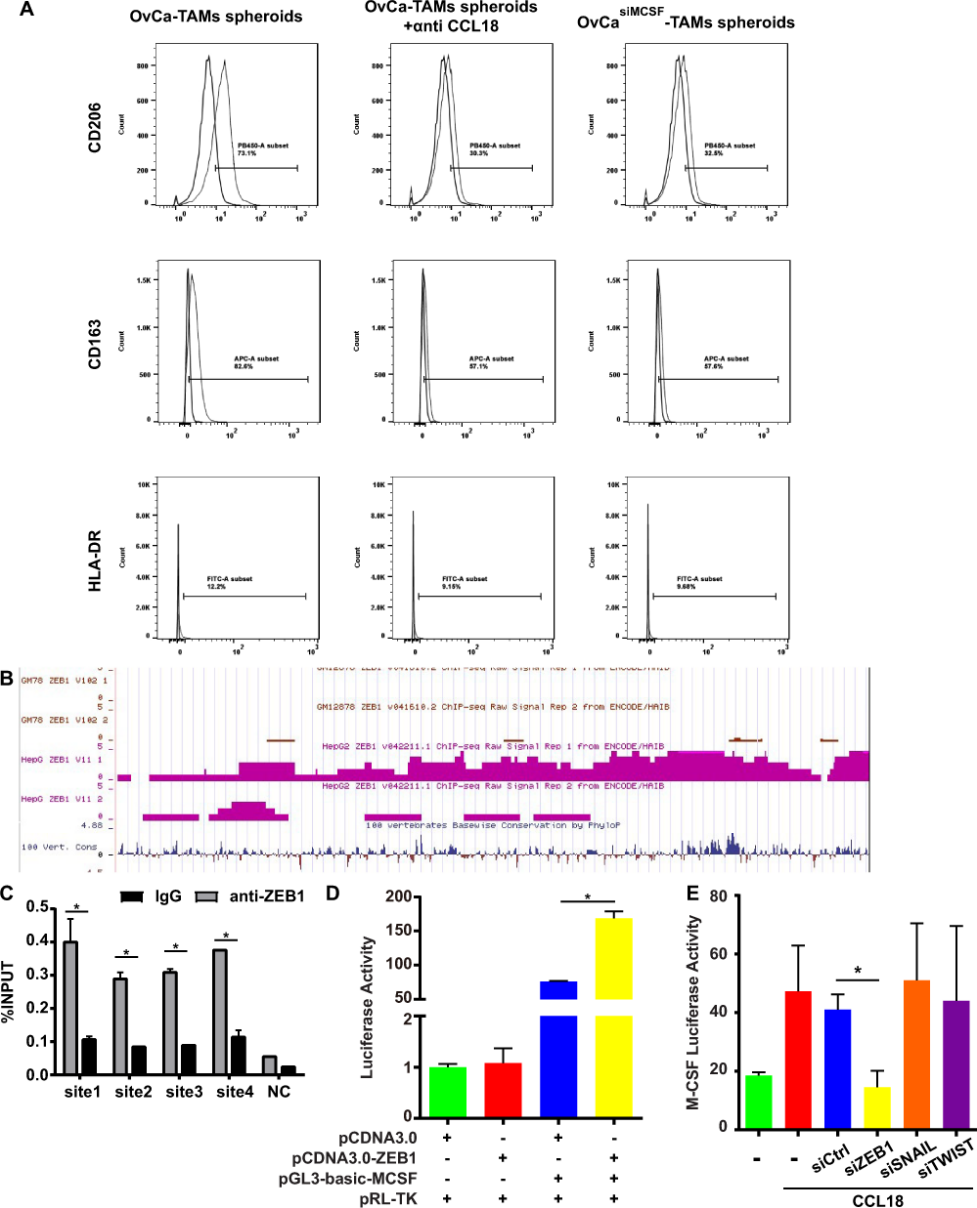
CCL18 induced the polarization of TAMs through the loop of CCL18-ZEB1-M-CSF in OvCa-TAMs spheroids. (A) FCM measured the percentage of M2-TAMs in the OvCa-TAMs spheroids, anti CCL18-treated OvCa-TAMs spheroids, and OvCa^siM-CSF^-TAMs spheroids. (B) The predictive ZEB1 transcription factor binding site on M-CSF by ENCODE data base. (C) Quantitative ChIP assays of M-CSF promoter regions were performed in SKOV3 using antibodies against ZEB1. IgG antibody was used as a negative control. (D) pGL3-basic-M-CSF and pRL-TK were co-transfected into SKOV3 with ZEB1 plasmid or the vector to measure the M-CSF activity after overexpression of ZEB1. Histogram indicated the data of luciferase activity measured 48 hours after transfection. (E) RLuc-MCSF-WT was transfected into SKOV3, CCL18-treated SKOV3, CCL18-treated SKOV3^vector^, CCL18-treated SKOV3^siZEB1^, CCL18-treated SKOV3^siSNAIL^, CCL18-treated SKOV3^siTWIST^, Luciferase activity was detected using the dual luciferase assay. Data were shown as mean±SD, and significant difference are indicated (*p<0.05, against control). CCL18, Chemokine (C-C motif) ligand 18; ChIP, chromatin immunoprecipitation; FCM, flow cytometry; M-CSF, macrophage colony-stimulating factor; OvCa, ovarian cancer; TAMs, tumor-associated macrophages; ZEB1, zinc finger E-box-binding homeobox 1.

The flow cytometry results show that the inhibition of CCL18 or M-CSF can impede the polarization of M2-TAMs in OvCa-TAMs spheroids (figure 4A). As to the putative TF of M-CSF, we focus on ZEB1, because it contains four M-CSF binding sites (figure 4B–D). To confirm whether ZEB1 binds the M-CSF promoter in vitro, chromatin immunoprecipitation experiments are carried out on SKOV3 cells. As shown in figure 4C, there is a rapid accumulation of ZEB1 protein in the promoter regions of M-CSF genes. Next, the luciferase assay shows that the activity of M-CSF is strongly increase after overexpression of ZEB1, suggesting that ZEB1 actives the promoter of M-CSF (figure 4D).

Finally, we intend to clarify the interplay among CCL18, ZEB1, and M-CSF. Luciferase results evidenced that CCL18 increased the M-CSF activity, no matter with or without the presence or absence of SNAIL and TWIST. Oppositely, the silence of ZEB1 abolished CCL18-induced activation of M-CSF. In summary, CCL18 upregulated the expression of M-CSF by increasing the expression of ZEB1, an important TF of M-CSF (figure 4E).

### ZEB1 in OvCa cells is responsible for the pro-tumoral phenotype and the formation of OvCa-TAMs spheroids

**Figure 5:**
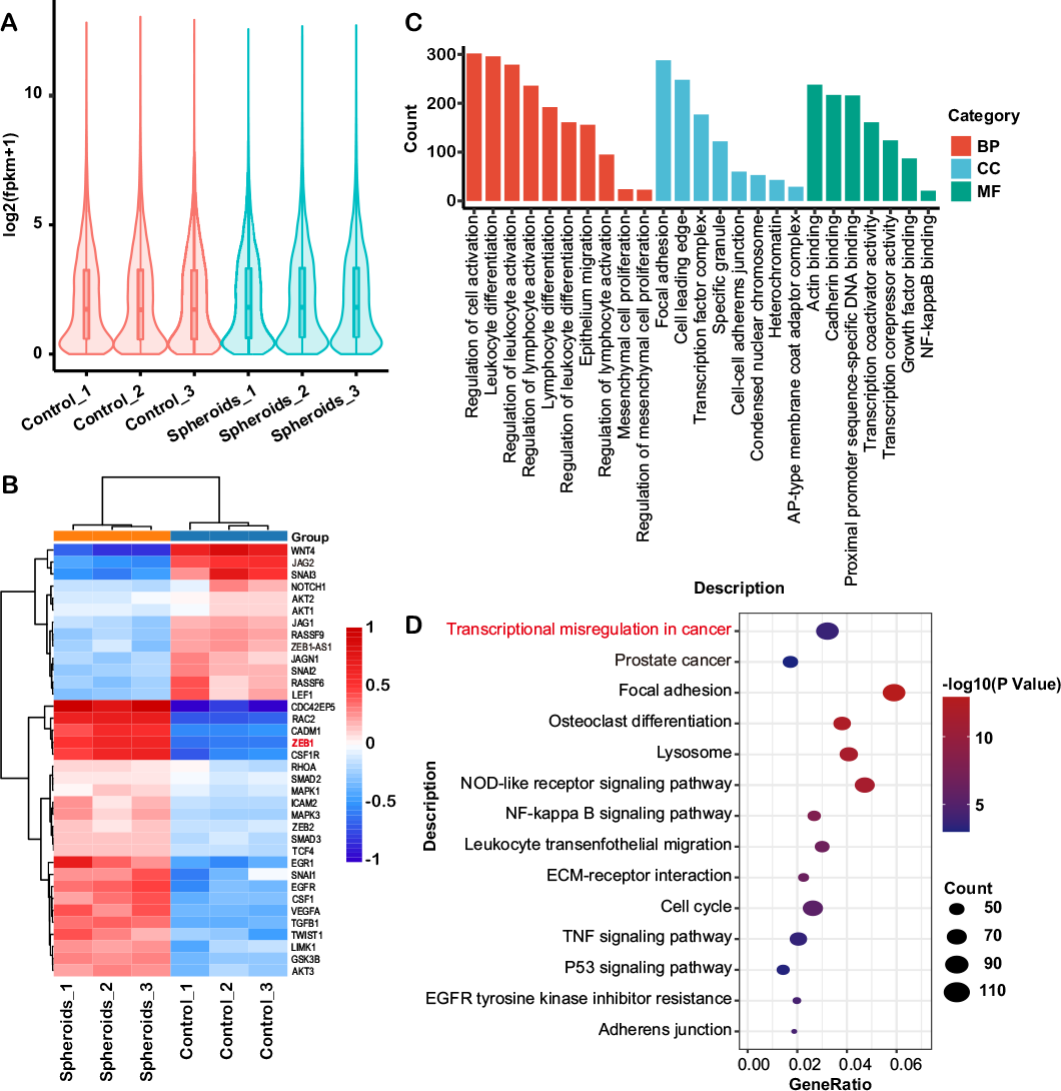
(A) Violin plots show the gene expression level of each sample, the width of each violin represents the number of genes under this expression level. (B) Hierarchical clustering of the differentially expressed genes associated with EMT in the OvCa cells and OvCa cells isolated from spheroids (p<0.05). Each group contains three samples. (C) GO classification of differential expressed genes in comparisons between OvCa cells and OvCa isolated from spheroids. The ordinate is the number of differentially expressed genes and the abscissa represents the GO term name. Orange represented biological process, blue represented cellular component and green represented molecular function. (D) KEGG pathway analysis revealed the enrichment of several important pathways among the differential expressed genes, including transcriptional misregulation in cancer and focal adhesion. EMT, epithelial-mesenchymal transition; GO, gene ontology; KEGG, Kyoto Encyclopedia of Genes and Genomes; OvCa, ovarian cancer; TNF, tumor necrosis factor.

RNA-seq was used to identify different gene expression between OvCa cells and OvCa cells from spheroids. In the violin plots, the overall shape and data distribution of the control group and spheroids group are similar, indicating the parallel samples in each group. In terms of the data volume, two groups are also comparable since the similar median and shape in the plots (figure 5A). The results showed that the expression levels of ZEB1 increased significantly in OvCa cells isolated from spheroids (figure 5B). The most enriched Gene ontology (GO) terms were annotated as actin binding in the molecular function category (GO:0003779, p=7.610570E-06, counts of DEGs=238), focal adhesion in the cellular compartment category (GO:0005925, p=1.787457E-19, counts of DEGs=288) and leukocyte differential in regard to the biological process category (GO:0002521, p=4.546801E-14, counts of DEGs=296) (figure 5C). Kyoto Encyclopedia of Genes and Genomes pathway analysis indicated that the transcriptional misregulation in cancer pathway (hsa05202, GeneRatio=0.032136, p=0.003641), focal adhesion pathway (hsa04380, GeneRatio=0.058981, p=2.7E-15) were significantly enriched (figure 5D).

**Figure 6:**
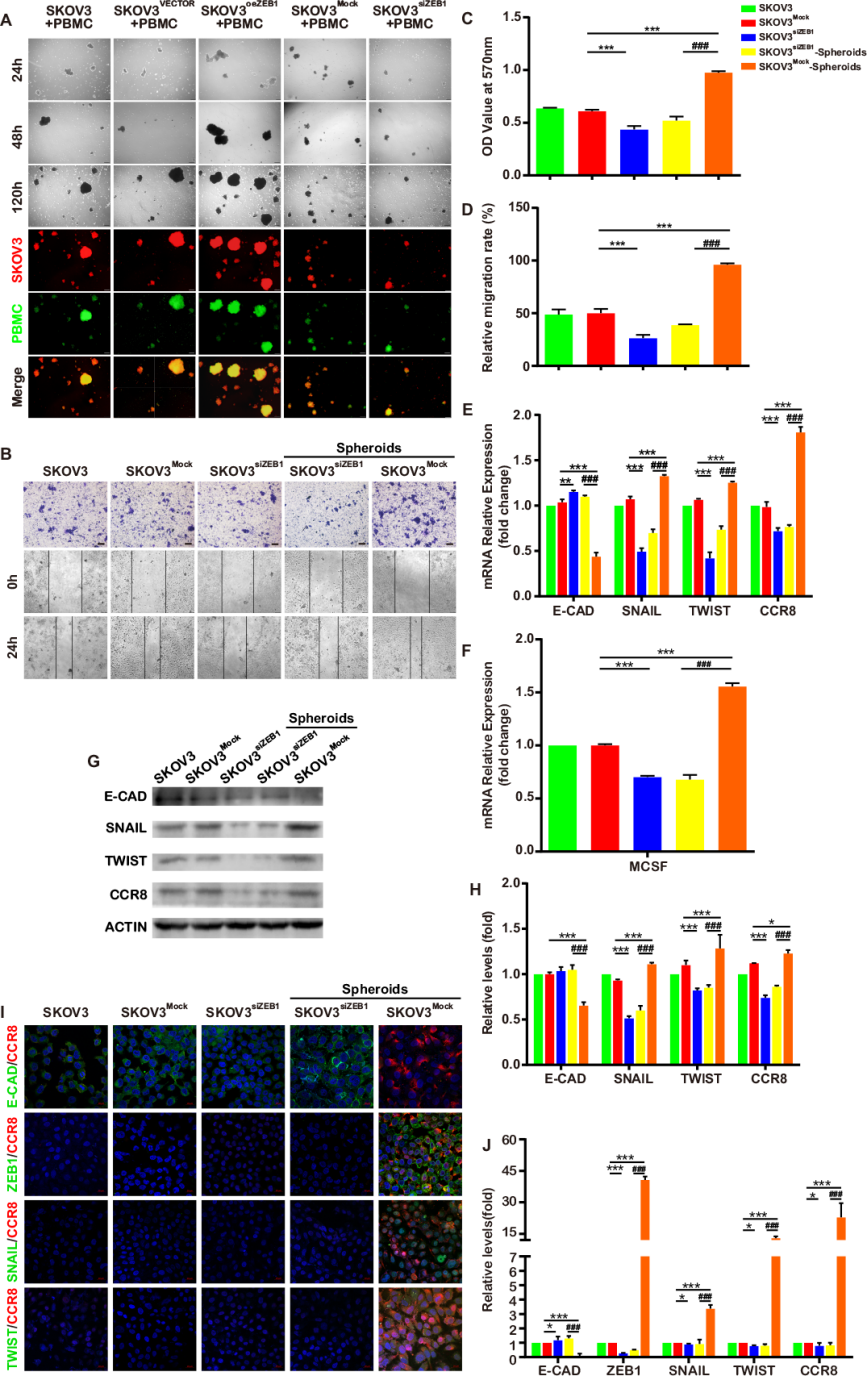
ZEB1 regulate the formation of OvCa-TAMs spheroids. (A) The formation of SKOV3-TAMs spheroids, SKOV3^oeZEB1^-TAMs spheroids and SKOV3^siZEB1^-TAMs spheroids in the 3D coculture system. (B) The invasive and migratory capacity of SKOV3 isolated from SKOV3-TAMs spheroids, SKOV3^oeZEB1^-TAMs spheroids and SKOV3^siZEB1^-TAMs spheroids were measured by transwell-migration assay and scratch wound healing assay. (C) The quantitation of transwell-migration assay for isolated SKOV3 (n=4 biologically independent samples per group and an average of five fields acquired from each sample). (D) The quantitation of scratch wound healing assay for isolated SKOV3 (n=4 biologically independent samples per group and an average of five fields acquired from each sample). (E–H) qRT-PCR and Western bot analysis the gene and protein expression of E-CAD, SNAIL, TWIST, CCR8 and M-CSF. (I, J) The immunofluorescence staining for E-CAD, SNAIL, TWIST and CCR8. All Data were shown as mean±SD, and significant difference are indicated (*p<0.05, **p < 0.01 and ***p<0.001 against control. ###p < 0.001 against SKOV3 with the knock down of ZEB1 in spheroid). CCR8, Chemokine (C-C motif) ligand 18; 3D, three-dimensional; E-CAD, E-cadherin; M-CSF, macrophage colony-stimulating factor; OvCa, ovarian cancer; PBMC, peripheral blood monocyte cells; qRT-PCR, quantitative reverse transcription-PCR; TAMs, tumor-associated macrophages; ZEB1, zinc finger E-box-binding homeobox 1.

These data showed a significant increase in expression of ZEB1 in the OvCa-TAMs spheroids. Therefore, we intend to assess the regulation of ZEB1 on the formation of OvCa-TAMs spheroids. In the case of 3D coculture of SKOV3 cells with macrophages, heterotypic spheroids composition of ovarian tumor cells and macrophages were formed. Cells in heterotypic spheroids remained viable and macrophages were located at the center of the spheroid. This has been previously reported for OvCa cells and proposed to be associated with the malignant phenotype of these cells that would therefore assemble around the macrophages.^21^ The number of SKOV3-TAMs spheroids per chamber increases with time. High dispersion, a phenomenon of cell proliferation, in the average diameter of spheroids is earlier found in SKOV3^oeZEB1^-TAMs spheroids than SKOV3^siZEB1^-TAMs spheroids. At the 120 hours, SKOV3^oeZEB1^-TAMs spheroids showed an increasing size and compactness degree, with tight accumulation of macrophages in the center, whereas SKOV3^siZEB1^-TAMs spheroids are still small with loose macrophages (figure 6, online supplemental figure 5, in green). These results indicated that the presence of ZEB1 of OvCa cells had major effects on the formation of spheroids.

Furthermore, we also investigated whether knocking down of ZEB1 can attenuate the regulation of TAMs toward OvCa cells in the spheroids. These evidences provide the fact that the migratory and invasive ability were decreased in the SKOV3^siZEB1^; moreover, after the cocultured with TAMs in the spheroids, the migratory and invasive ability of SKOV3^siZEB1^ could not be increased (figure 6B–D, online supplemental figure 6). In additional, the expression of mesenchymal markers, CCR8 and M-CSF in the SKOV3^siZEB1^ also decreased, which seldom be rose by coculturing with TAMs in the spheroids (figure 6E–J). These phenomenons have been also proved in HO-8910, a kind of human epithelial ovarian cance cell line (online supplemental figure 7). It is reasonable to suspect that the silence of ZEB1 not only decrease the malignancy of OvCa cells, but also impede the pro-tumoral effects of TAMs toward OvCa cells in the spheroids, which provides the evidence for ZEB1 to be used as a therapeutic target for OvCa.

### OvCa-TAMs spheroids from ascites are more tumorigenic in vivo, and OvCa^oeZEB1^ cells promote transcoelomic metastasis

**Figure 7:**
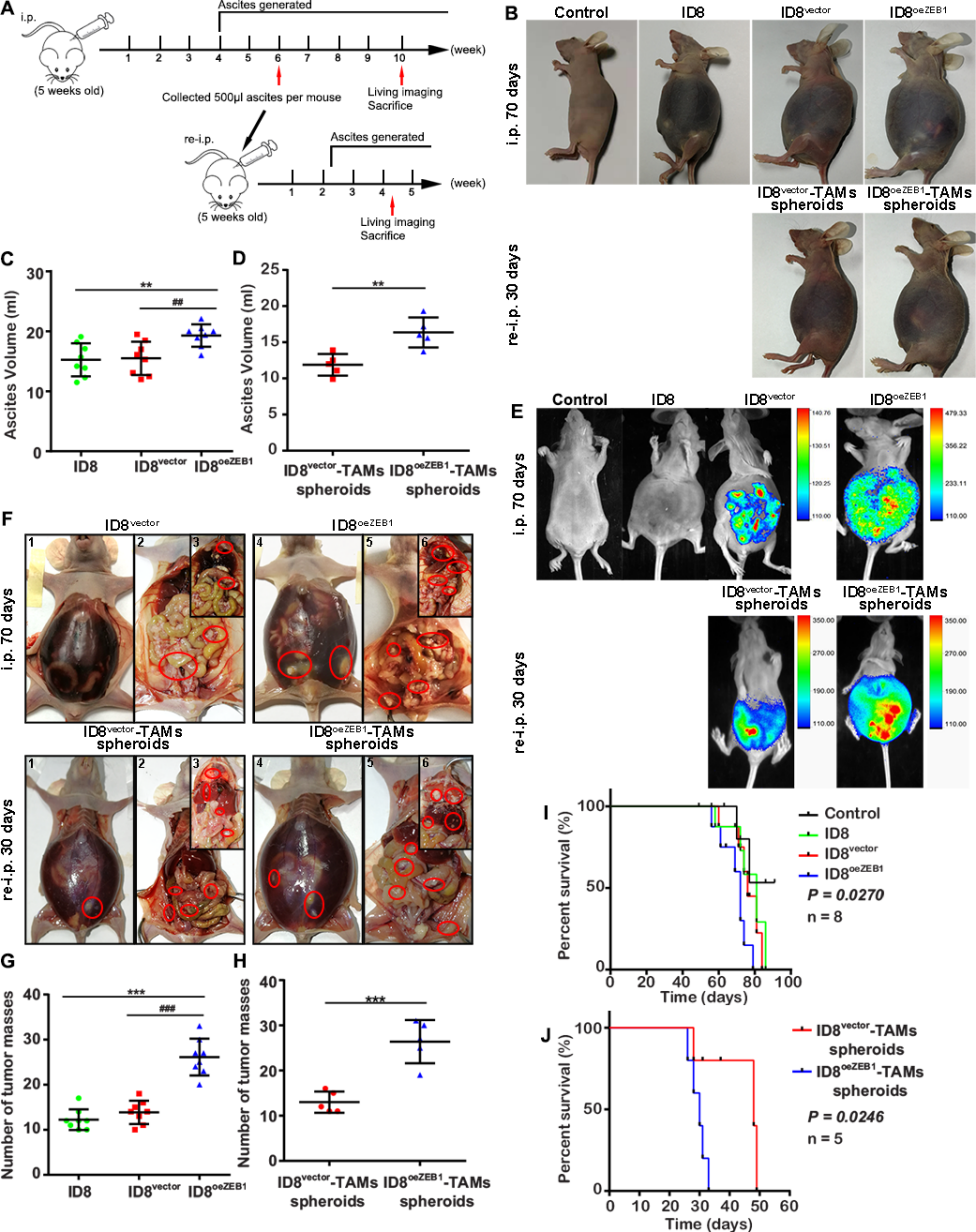
TAMs-OC spheroids induce the early transcoelomic metastasis. (A) Schematic depiction of our in vivo experimental design. Nude mice received intraperitoneal injections of ID8 cells to construct OvCa mice model. At the sixth week, the OvCa-TAM spheroids were collected form these OvCa mice and then re-injected into new nude mice. At the 10th week, the OvCa mice were used to perform the in vivo imaging, and then were sacrificed to analyze their bleeding tendency ascites, tumor burden and metastatic tumor nodules. The re-injected mice were used to perform the in vivo imaging, and then were sacrificed to analyze their bleeding tendency ascites, tumor burden and metastatic tumor nodules at the fourth week. (B) Representative images of the bleeding tendency ascites in the first injection groups including control, ID8^vector^, ID8^oeZEB1^ group; and TAMs-OC spheroids reinjection groups, including ID8^vector^-TAMs spheroids and ID8^oeZEB1^-TAMs spheroids (n=5 mice per group). (C, D) Ascitic fluid volumes from first injection groups at the 70 days after injection and TAMs-OC spheroids reinjection groups at the 30 days after injection. (E, F) To analyze the metastatic symptoms, on day 70 after tumor implantation and day 30 after TAMs-OC spheroids implantation, imaging with luciferase and imaging with metastatic colonization in tumor bearing BALB/c-nu mice. (G, H) Representative images and statistical analysis of tumor implantation in peritoneum and mesentery. (I) Kaplan-Meier curves indicating the survival of ID8, ID8^vector^, ID8^oeZEB1^ tumor-bearing BALB/c-nu mice (n=8 mice per group). (J) Kaplan-Meier curves indicating the survival of ID8^vector^-TAMs spheroids, ID8^oeZEB1^-TAMs spheroids tumor-bearing BALB/c-nu mice (n=5 mice per group). Data are presented as mean±SD, unpaired two-sided Student’s t-test with no correction for multiple comparison, **p < 0.01 and ***p < 0.001 against control. ##p < 0.01 and ###p < 0.001 against ID8 vector group. OvCa, ovarian cancer; TAMs, tumor-associated macrophages.

It has been reported that the formation of spheroids promotes OvCa cells growth and escaping from immune attack.^22^ Based on our above studies, we suggested that there is a ZEB1 driving loop between OvCa cells and TAMs in the ascites spheroid, which improves the malignancy of OvCa cells. The in vivo study was used to assess the effects of OvCa-TAMs spheroids on transcoelomic metastasis. In comparison with ID8, intraperitoneal injection of ID8^oeZEB1^ induced the much more spheroids in ascites (online supplemental figure 8A, B) and serious metastatic symptoms, including bleeding tendency ascites, higher tumor burden and metastatic tumor nodules at the 70th day after injection (figure 7B, C, E and F). Accordingly, mouse survival rate was decreased by upregulating the expression of ZEB1 in the OvCa (figure 7I). These results demonstrated that ZEB1 promotes OvCa transcoelomic metastasis.

Next, the effects of OvCa-TAMs spheroids on transcoelomic metastasis were also measured. We isolated ID8^vector^-TAMs spheroids and ID8^oeZEB1^-TAMs spheroids from above ID8^vector^ mice model and ID8^oeZEB1^ mice model, and then intraperitoneal injected them into new BALB/c-nu mice, named as ID8-TAMs spheroids mice model. The bleeding tendency ascites, tumor burden and metastatic tumor nodules were appeared in ID8-TAMs spheroids mice model on 30 days after injection, which was approximately 40 days earlier than ID8 mice (figure 7G, H). Survival rate was also reduced in both spheroids mice model (figure 7I). Thus, OvCa-TAMs spheroids can accelerate the process of transcoelomic metastasis. Of notes, ID8^oeZEB1^-TAMs spheroids mice showed much more serious metastatic symptoms (figure 7G, H) and decreasing survival rate than ID8^vector^-TAMs spheroids mice (figure 7I). Consequently, OvCa-TAMs spheroids is critical to improve OvCa transcoelomic metastasis, and over expression of ZEB1 can accelerate spheroids’ effects.

## Discussion

An interaction of TAMs with OvCa has been observed in both mouse models and human patients. Elimination of peritoneal macrophages, but not other lymphocyte cells, inhibit peritoneal metastasis.^23^ Previous studied have indicated that macrophages promote OvCa peritoneal transcoelomic metastasis through forming spheroids.^9 24^ In this study, we compared the interactions of TAMs and OvCa cells in the spheroids and in the disaggregated state, and investigated the molecular mechanism whereby the positive feedback loop (CCL18-ZEB1-M-CSF) to promote the growth and metastasis of OvCa in spheroids. In detail, OvCa cells with mesenchymal feature can release M-CSF to polarize M2 subtype. In turn, M2-TAMs secrete CCL18 to induce EMT of OvCa cells by increasing the expression of ZEB1 and other EMT-TFs, and then ZEB1 promote the transcription of M-CSF. Given that ZEB1 is significance for this feedback loop in OvCa-TAMs spheroids. The inhibition of ZEB1 in OvCa cells not only can decrease the OvCa-TAMs spheroids’ formation, but also impede the regulation of TAMs toward OvCa cells. In summary, this work has elucidated the causality and mechanisms of tumorigenic activities of the loop: CCL18-ZEB1-M-CSF and open our understanding of OvCa-TAMs spheroids in increasing transcoelomic metastasis.

Many studies believed that the formation of spheroids is beneficial to transcoelomic metastasis of ovarian tumors, because spheroids offer an environment to protect anoikis and induce the proliferation of OvCa cells.^25–27^ Indeed, the different interactions between TAMs and OvCa cells in the spheroid and in the disaggregated state are unknown. Our study confirmed that TAMs in the spheroids increased the malignancy of surrounding OvCa cells, but these effects were minor in the disaggregated state. Similarly, OvCa cells in the spheroids polarized approximate 80% macrophages into M2 subtype, but OvCa cells in the disaggregated state only induced about 10% M2 subtype. These differences are possibly due to specific signal pathway in the spheroids. Some signal pathways have already been studied in the spheroids. For example, tumor metabolic studies implied that spheroid OvCa cells had metabolic properties of antiapoptosis and aggressive proliferation by regulating the glucose cycle into pentose cycle.^28^ A further study showed that TAMs expressed integrin to recognize its receptor intercellular cell adhesion molecule-1 on the OvCa cells to form a tight spheroid to induce the growth of OvCa cells.^9^ Thus, we suggest that the formation of spheroid should not be considered as a simply spatial aggregation of tumor cells, actually it offers a specific signal pathway to confer more invasiveness for tumor cells.

EMT processing increases tumor cell aggressive property and enriches cancer stem cells (CSCs), a kind of tumor cells with stem-like character, tumor heterogeneity and the most lethal features.^29^ Our results find that spheroidal OvCa cells around TAMs endowed full range of mesenchymal phenotype, but disaggregated OvCa cells cocultured with TAMs only showed partial mesenchymal markers or even expressed epithelial marker. Actually, at the different tumor state, the heterogeneous nature of the EMT program has been showed. Study indicated that tumors at the invasive front express the properties of mesenchymal phenotype as the EMT process is performed, whereas, primary tumors still maintain the epithelial phenotype.^30–32^ Therefore, it is possibly an EMT gradient mechanism during tumor cells, like full EMT to partial EMT, and no EMT program, depending on the metastatic state of tumors.^33^ As to the circulating tumor cells (CTCs), a kind of metastatic tumor cells in the blood, showed fully mesenchymal phenotype in a triple-negative molecular subtype (estrogen receptor−/progesterone receptor−/human epidermal growth factor 2−) breast cancers.^34^ But some CTCs in other kinds of cancers have epithelial markers and mesenchymal markers at the same time, implying that an EMT process is going on during the metastases of tumor cells.^35 36^ Interestingly, a study showed that inflammation is necessary for the circulation entry of cancer cells by inducing EMT.^37^ Our results about ascitic OvCa cells are consistent with the studies of CTCs. Due to the chemical gradient, TAMs can strongly induce the EMT program on surrounding OvCa cells in spheroids, but the effects are decreased between the disaggregated TAMs and OvCa cells. Therefore, the formation of OvCa-TAMs spheroids is a powerful step to improve the metastatic ability of OvCa cells. The gradient mechanism of EMT process is regulated by the intrinsic metastatic heterogeneity inducing from different signaling cross-talk or various mutation profiles.^38^ Although the importance of OvCa-TAMs spheroids in transcoelomic metastasis has been well discussed above, an in-depth mechanism regarding which signal pathway regulated OvCa cells and TAMs in spheroids is largely unknown. Previous studies reported the increasing number of M2-TAMs have been considered as a reason of poor patient prognosis in various cancer types, such as breast, lung, ovarian and prostate, as M2-TAMs promote the EMT process in carcinoma cells by producing chemokine.^39 40^ A further study showed elimination of macrophages from teratocarcinoma allografts mice bring about the increasing epithelial carcinoma cells and decrease it in mesenchymal carcinoma cells.^41^ Our in vitro study indicated that macrophages in the spheroids secreted much higher level of CCL18 than in the transwell. The neutralization of CCL18 or its receptor CCR8 in the spheroids, diminished the mesenchymal features of OvCa cells. In addition, a panel of EMT markers, including TWIST, SNAIL, and ZEB1, were significantly activated as CCL18 target genes. Actually, CCL18 has been proven to induce EMT process in breast tumor cells and gastric tumor cells.^42^ Therefore, TAMs in the spheroids induced mesenchymal features of surrounding OvCa cells through CCL18 signal pathway. Previous study showed proinvasive tumor cells fully expressed ZEB1 to obtain stem-like phenotype. Thus, high expression of ZEB1 related to a poor prognosis in most cancers, including ovarian carcinoma.^43^ *Marlies Cortés* indicated that ZEB1 increased the interaction between cancer cells and macrophages by combining to the regulatory regions of target gene.^44^ Our results showed only ZEB1 was responsible for M2-TAMs polarization by binding to M-CSF, which is a key macrophage-lineage cytokine to induce the functional M2-TAMs in metastases environment.^19^ As a result of that, OvCa cells in the spheroid secreted abundant of M-CSF to promote the polarization of M2-TAMs. Comparison with OvCa-TAMs spheroids, the interactions between disaggregated OvCa cells and TAMs are not as strong as in spheroids, because the CCL18-ZEB1-M-CSF loop is absence.

The role of ZEB1 in regulation of the formation of OvCa-TAMs spheroids, an early step prior to peritoneal metastatic colonies, has not been previously discussed. Previous studies shed light that the increased expression and functional induction of EMT-TFs, the master regulators of EMT process, are able to coordinate various transcriptional alterations relate to EMT process. EMT-TFs groups mainly include SNAIL family, TWIST family and ZEB1 family.^45^ Among them, only EMT-TF ZEB1 can induce both CSCs and mesenchymal features,^46^ while others inhibit the activation of CSC-like phenotype.^47–49^ As to mouse model of pancreatic ductal adenocarcinoma, individual knockout of either Twist1 or Snai1 seldom influences the metastatic development,^50^ but silence the expression of ZEB1 effectively inhibit metastatic colonization and CSC feature.^46^ These results imply that EMT-TFs have overlapping effects to activate EMT, nonetheless the specific biological functions that are regulated by EMT-TFs are different. Our results found that the expression level of ZEB1 in the OvCa cells positive correlated to the formation of OvCa-TAMs spheroids. In additional, silence ZEB1 impede the TAMs-induced EMT process of OvCa cells in the spheroids. Our in vivo study also supported those results. In comparison with the ID8-induced OvCa mice model, ID8^oeZEB1^-constructed OvCa mice models show increased number of OvCa-TAMs spheroids, and a faster and worse metastatic process. Furthermore, in comparison with ID8-injected ovarian mice, the ID8-TAMs spheroids-injected ovarian mice showed the faster metastatic colonization and poorer survival. The metastatic colonization 40 days earlier in the ID8-TAMs spheroids-injected ovarian mice than ID8-injected ovarian mice. In addition, the survival of ID8-TAMs spheroids-injected mice were greatly decreased than ID8-injected mice, suggesting that ZEB1 plays an important role in promotion in promotion of inhibition of the form of ID8-TAMs spheroids could impede the OvCa metastasis at the early stage. Furthermore, ID8^oeZEB1^-TAMs spheroids isolated from ID8^oeZEB1^-bearing mice resulted in the faster metastasis and poorer survival than ID8-TAMs spheroids, indicating that the ZEB1 not only regulate the formation of OvCa-TAMs spheroids, but also increase the metastatic ability of OvCa-TAMs spheroids. Suppression of ZEB1 in OvCa cells possibly can offer an alternative approach for therapy of OvCa transcoelomic metastasis.

Taken together, our study proves the formation of OvCa-TAMs spheroids is an important process of early ovarian transcoelomic metastasis, as in which a specific feedback loop CCL18-ZEB1-M-CSF can induce the polarization of M2-TAMs and the EMT process on OvCa cells. Moreover, our study also demonstrates that ZEB1 functions as a positive regulator of the formation of OvCa-TAMs spheroids, thereby the expression level of ZEB1 directly affects the transcoelomic metastasis and prognosis. Our study has revealed the underlying pro-tumoral effects of OvCa-TAMs spheroids, suggesting a therapeutic method to impede the transcoelomic metastasis and improve the outcome of ovarian patients.

## Data Availability

Data are available in a public, open access repository.
