# Correction: Tumor-associated macrophages induced spheroid formation by CCL18-ZEB1-M-CSF feedback loop to promote transcoelomic metastasis of ovarian cancer

**DOI:** 10.1136/jitc-2021-003973corr1

**Published:** 2022-04-20

Long L, Hu Y, Long T, *et al*. Tumor-associated macrophages induced spheroid formation by CCL18-ZEB1-M-CSF feedback loop to promote transcoelomic metastasis of ovarian cancer. *J Immunother Cancer* 2021;**9:**e003973. doi:10.1136/jitc-2021-003973

This article has been corrected since it was first published online. Min Wang has been added as co-corresponding author, and the affiliations and contributorship statement have been updated accordingly.

