# Supplementary material for: Tumor-associated macrophages induced spheroid formation by CCL18-ZEB1-M-CSF feedback loop to promote transcoelomic metastasis of ovarian cancer
Source: J Immunother Cancer. 2021 Dec 25;9(12):e003973. doi: 10.1136/jitc-2021-003973 (PMC8718465; doi:10.1136/jitc-2021-003973)
Supplement: Supplementary data [file jitc-2021-003973supp001.pdf]

Supplemental materials

Tumor-associated macrophages induced spheroid formation by CCL18-ZEB1-M-CSF feedback loop to promote transcoelomic metastasis of ovarian cancer

Lingli Long[1*#], Yue Hu, [1*] Tengfei Long,[2*] Xiaofang Lu,[3*] Ying Tuo,[4] Yubing Li,[5] ZunFu Ke[6#]

Author affiliations

[1]Translation Medicine Center, The First Affiliated Hospital, Sun Yat-Sen University, Guangzhou, China, 510080; huyue29@mail2.sysu.edu.cn (Y.H.); longll@mail.sysu.edu.cn (L.L.L)

[2]Department of Gynaecology and Obstetrics, Sun Yat-Sen Memorial Hospital, Sun Yat-Sen University, Guanghzou, China, 510120; longtengfei811@163.com (T.F.L)

[3]Department of pathology, The Seventh Affiliated Hospital, Sun Yat-Sen University, Shenzhen, China, 518107; zdluxiaofang2003@163.com (X.F.L.)

[4]Department of pathology, The First Affiliated Hospital, Sun Yat-Sen University, Guangzhou, China, 510080; tuoying@mail.sysu.edu.cn (Y.T.)

[5]The Reproductive Center, The First Affiliated Hospital, Sun Yat-Sen University, Guangzhou, China,510080; liybin@mail.sysu.edu.cn (Y.B.L.)

[6]Molecular Diagnosis and Gene Testing Center, The First Affiliated Hospital, Sun Yat-Sen University, Guangzhou, China,510080; kezunfu@mail.sysu.edu.cn. (Z.F.K)

[*] Lingli Long, Yue Hu, Tengfei Long and Xiaofang Lu contributed equally.

[#]Correspondence:

Pro. ZunFu Ke, kezunfu@mail.sysu.edu.cn.

Dr. Lingli Long, longll@mail.sysu.edu.cn

Materials and Methods

Clinical samples

Ascitic fluid collection were collected from consenting patients with advanced stage (i.e., stages III–IV) ovarian cancer (OvCa). After collected by using Falcon™ cell strainers from ascitic fluid, tumor spheroids were transferred to a 50 ml conical centrifuge tube, and washed for 3 times by medium (0.5% BSA with PBS 10 ml) for 2 min. At the end, the suspension was centrifuged at 200 g for 3 min (room temperature). After centrifugation, human tumor spheroids were harvested and used for sectioning and staining. Ovarian cancer tissues and ascitic fluid were obtained from the Department of Obstetrics and Gynecology, The first affiliated hospital of Sun Yat-sen University. All protocols have been approved by IEC for clinical research and animal trials of The first affiliated hospital of Sun Yat-sen University (NO.2021-483). All patients have given their written informed consent.

Cell culture and treatments

Human ovarian cancer adenocarcinoma cell line SKOV3 and mouse ovarian cancer cell line ID8 cells were obtained from the cell bank of Chinese Academy of Science (Shanghai). Peripheral blood monocyte cells (PBMC) were isolated from volunteers' peripheral blood by Ficoll-Paque density gradient centrifugation.

Ovarian cancer cells (SKOV3 and ID8) were cultured in DMEM (#11965-092,

Gibco, USA) supplemented with 10% FBS (#10091-148, Gibco, USA) and 1% penicillin-streptomycin (#15140-122, Gibco, USA). PBMC were maintained in RPMI 1640 medium (#61870-036, Gibco, USA) containing 10% FBS and 1% penicillin-streptomycin. Then, PBMC were induced by 20 ng/ml M-CSF (HY-P7050, MCE, USA) for 72 h and differentiated to M2-like macrophage using 20 ng/ml IL-4 (HY-P70445, MCE, USA) for 48 h. All the cells were incubated at 37 °C in an atmosphere with 5% CO_2.

ZEB-1 knockdown and overexpression

Ovarian cancer cells were seeded into 12-well culture plates and incubated overnight to 70% confluence. ZEB1 overexpressed plasmid (CMV-MCS-3FLAG-SV40-Neomycin and CMV-MCS-3FLAG-SV40-Neomycin-ZEB1) and small interfering RNA (siRNA-Mock and siRNA-ZEB1) were respectively infected in cells. The cells were cultured with Opti-MEM (#31985-088, Gibco, USA) using the enhanced infection solution for 4h, after which the medium was replaced with DMEM containing 10% FBS. Besides, lentiviral vector GV260-ZEB1-Luciferase and GV260-Luciferase were used for the ZEB1 overexpression in ID8 cells. After transfection, DMEM containing 10% FBS and puromycin (BS111-25, Biosharp, China) was used to screen the stable transfected cells. Infection effieciency was calculated by real-time PCR and Western blot.

Tumor cell co-culture with macrophages

In transwell co-culture system, macrophages were seeded in transwell insert and tumor cells were in the lower chamber of 6-well transwell apparatus. Total number of cells was 2 × 10^5 per well and the ratio of SKOV3 and M2-like macrophage was 10:1.

For spheroidization, total number of cells in ultra-low-attachment 6-well plates was 2 × 10^5 per well and 5 × 10^3 cells per well in ultra-low-attachment 96-well plates. The ratio of SKOV3 and M2-like macrophage also was 10:1. Tumor cells and macrophages were pre-stained with DeepRed and CMFDA (M22426 and C7025, Invitrogen, USA) respectively. Spheroids' formation was observed daily through an inverted fluorescence microscope.

After 120 h co-culture, spheroids were collected by centrifugation and harvested by treatment with 10% trypsin-EDTA. At the end of the incubation period, spheroids should be almost completely digested and no longer visible. Magnetic label CD14 was used as a macrophage marker. After incubated in 4 °C for 15 minutes, using an appropriate magnetic activated cell sorting (MACS) column and MACS Separator to separate the TAMs ($CD14^+$ cells) and OvCa cells ($CD14^-$ cells).

FCM

After co-culture with ovarian cancer cell line, PBMC suspensions were harvested by treatment with 10% trypsin-EDTA, and detached cells were washed with cold PBS. The cells were labeled with FITC-CD11b, PE-CD163, BV421-CD163 and

APC-HLA-DR antibodies (562793, 556018, 564062 and 559869, BD Biosciences, USA) for 15 minutes on ice. Isotype antibody served as a negative control. Flow cytometry was performed on a FACSCalibur (BD Biosciences, USA). The data were analyzed with FlowJo software.

Migration assay and Wound healing assay

The migration activity of tumor cells was demonstrated using the Transwell cell culture system (Cornning; pore size, 8 μm) *in vitro*. To test the migration activity of SKOV3 cells after co-culture, 1 × 10^4 SKOV3, $SKOV3^{Transwell}$ and $SKOV3^{Spheroids}$ cells were seeded on the upper chamber and M2-like macrophages were placed in the lower chamber of 12-well transwell apparatus. After 12 h of incubation, SKOV3 cells on the upper face of the Transwell membrane were removed with a cotton swab. The cells on the bottom surface of the Transwell membrane were then fixed in 4% paraformaldehyde and quantified by Crystal Violet staining: Migrated cells were counted in 5 randomly chosen fields under a microscope (200×). Then, stain was dissolved in 33% ethylic acid and O.D. measured at 570nm on a microplate reader (SynergyH1, BioTek, USA). To test the proliferation activity, SKOV3, $SKOV3^{Transwell}$ and $SKOV3^{Spheroids}$ cells were seeded in the 96-well culture plates and incubated overnight to 100% confluence. The confluent cells were scratched with a 200 μl pipette tip and then plates were washed with PBS to remove non-adherent cells. Wound area was determined using an inverted microscope after 24 h.

Cytokines Array

Raybio® Human Cytokine Antibody Array G6 was performed according to the manufacturer's protocol (Raybiotech, Inc.). After co-culture for 48 h and incubation in FBS-free medium for an additional 48 h, supernatants were collected by centrifugation at 2000 × g for 10 min, and were tested by the protein array company (H-Wayen Biotechnologies, Shanghai). Agilent SureScan Dx Microarray Scanner was used for scanning, and GenePix Pro 6.0 software was used to read the original data of chip images obtained by scanning. During data analysis, Positive Control was used to normalize the signal values between samples, and finally, the normalized data were used for comparison between groups.

Chromatin immunoprecipitation (ChIP)

After transfected with ZEB1 overexpression plasmid, cells were fixed with 1% (v/v) formaldehyde and crosslinking was stopped with 1.25 M glycine (G8790, Sigma, Germany). Chromatin was digested with Micrococcal Nuclease (CS0004, Sigma, Germany) into 150-900bp DNA-protein fragments. The complex was then co-precipitated with control IgG or antibodies against ZEB1 and was captured by protein A/G magnetic beads. Then the crosslinks were reversed and DNA was purified and ready for analysis. Samples were tested by CookGEN Biotechnologies (Guangzhou).

Luciferase assay

In six-well plates, SKOV3 cells were cultured to approximately 70% confluence and then co-transfected with luciferase vector and either siRNAs or negative control. After 48 h cultured with recombined human CCL18, luciferase assay extracts were prepared using the Dual Luciferase Reporter Assay System kit (Promega). Luciferase activity was normalized to the Renilla activity. Data was read by a microplate reader (SynergyH1, BioTek, USA).

RNA-seq

After RNA quantification and qualification, a total amount of 1 μg RNA per sample was used as input material for the RNA sample preparations. Sequencing libraries were generated using NEBNext® UltraTM RNA Library Prep Kit for Illumina® (NEB, USA) following manufacturer's recommendations and index codes were added to attribute sequences to each sample. Differential expression analysis of two groups (three biological replicates per condition) was performed using the DESeq2 R package (1.16.1). DESeq2 provide statistical routines for determining differential expression in digital gene expression data using a model based on the negative binomial distribution. The resulting P-values were adjusted using the Benjamini and Hochberg's approach for controlling the false discovery rate. Genes with an adjusted P-value <0.05 found by DESeq2 were assigned as differentially expressed (DEGs). We performed Gene ontology (GO) enrichment analysis of the

DEGs was implemented by the clusterProfiler R package, in which gene length bias was corrected. GO terms with corrected P-value less than 0.05 were considered significantly enriched by DEGs. Kyoto Encyclopedia of Genes and Genomes (KEGG) is a database resource for understanding high-level functions and utilities of the biological system, such as the cell, the organism and the ecosystem, from molecular-level information, especially large-scale molecular datasets generated by genome sequencing and other high-through put experimental technologies (http://www.genome.jp/kegg/). We also used clusterProfiler R package to test the statistical enrichment of DEGs in KEGG pathways.

Animal models

The female BALB/c nude mice (age 5-week) were purchased from laboratory animal center of SYSU. Mice were maintained in a light/dark cycle with free access to food and water, room temperature (25 °C), in 40–60% of humidity. PhD students qualified in animal experiments and full-time junior technicians from the animal laboratory center are responsible for animal caretaking. To prove the character of ZEB1 in peritoneal spread of ovarian cancer, nude mice were anesthetized with isoflurane by an inhalation anesthesia machine (KW-MZJ-4, KEW BASIS company, China), and then an orthotopic mouse model was established by injecting 3 × 10^6 mouse ID8 OCs into the peritoneal cavities of nude mice. If narcotic drugs overdose, emergency treatment should be immediately performed. If the first aid fails, the mouse death is inevitable and should be euthanized. If insufficient drug dose leads to

insufficient anesthesia depth in mice, drug dose should be increased in time to avoid pain in mice. Mice were randomly divided into Control, ID8, ID8^{VECTOR}-Luc and ID8^{oeZEB1}-Luc groups (n = 16 each group; half of them for survival analysis). Ascites was beginning to generated at the 4th weeks after injection. In the 10th week, mice were injected 75 mg/kg D-luciferase (ab228546, Abcam, UK) and observed by in vivo imaging system (eXplore Locus, GE Healthcare, USA). The mice were euthanasia by use of CO_2 at 30% chamber replacement rate, ascites fluid volume and location of tumor nodules were recorded in model mice. The spheroids were isolated from the ascites of ID8^{VECTOR}-Luc and ID8^{oeZEB1}-Luc ovarian mice, and injected in to other twenty nude mice to establish new ovarian mice models (half of them for survival analysis). A metastasis model was also established after intravenous injection 2 × 10^5 ID8^{VECTOR}-Luc or ID8^{oeZEB1}-Luc (n = 5 each group). The mice were sacrificed after 8 weeks and collected the lung tissues. Isolated tissue was fixed with 4% paraformldehyde for 2 days and embedded in paraffin. HE staining permits the visualization of the morphology and cellular heterogeneity of the tissue.

Immunofluorescence staining

OvCa cells after co-culture system were fixed with 4% paraformaldehyde for 10 min at room temperature. To block the nonspecific binding, the cells were incubated with 3% BSA in PBS for 1 h. And then, incubated with mouse monoclonal antibodies E-CAD (#13-1700, invitrogen, USA), ZEB1 (ab181451, Abcam, UK), SNAIL (#14-9859-82, invitrogen, USA) and TWIST (#MA5-32927, invitrogen, USA) at 4°C

for 24 h. After washing with PBS three times, the cells treated with 0.1% Triton X-100 for 10 min or not. Rabbit polyclonal antibodies against cytokeratin 7 (CK7) (#4898, CST, USA) or chemokine receptor 8 (CCR8) (ab63772, Abcam, UK) were incubated at 4°C for 24 h after washing. And finally, cells were incubated with Alexa Fluor 594-conjugated anti-rabbit IgG (#8760, Invitrogen, USA) and Alexa Fluor 488-conjugated anti-mouse IgG (#3655, Invitrogen, USA) for 1h at room temperate. Nucleic were visualized with DAPI. Images were acquired on a Zeiss confocal microscope.

Immunohistochemical staining

Tumors from patients were fixed in 4% paraformaldehyde, embedded in paraffin and then sliced into 5 μm thick sections. After being deparaffinized, slides were incubated with primary antibody against CD206 (ab8918, Abcam, UK), CD68(#76437, CST, USA), CK7 (#4898, CST, USA) and PITPNM3 (PAB6609, abnova， CHINA), and then incubated with a secondary antibody. Diaminobenzidine (DAB) Kit (DA1016, Solarbio, CHINA) was used to visualize immunoreactive proteins. The protein levels were detected using Polink-2 Plus IHC Detection System. The stained slides were observed under the light microscope.

RNA extraction and quantitative Real-Time PCR (qRT-PCR)

RNA was isolated from tumor cells and macrophages with TRIzol reagent and the RNeasy Plus Mini Kit (#R2071, ZYMO RESEARCH, USA). cDNA was

synthetized by utilizing the QuantiTect Rev. Transcription Kit (4368814, Applied Biosystems) following the manufacturer's instructions. Real-time PCR was performed using SYBR Green PCR Master MIX (DBI-2043, DBI, Germany). The PCR reaction parameters were:2 min at 95°C, followed by 40 cycles of 10 s at 95°C, 30 s at 56°C and 30 s at 72°C. Melting curve parameters were: 1 min at 95°C, 1min at 55°C and followed by 0.5°C/cycle at 55-95°C. Data were calculated using $2^{-\Delta\Delta CT}$ method. The sequence of primers is listed in the supplemental table 2.

Western blot

Cells were harvested and homogenized in the RIPA lysis buffer (#20-188, Sigma, Germany). The lysates were centrifuged at 13,000 g for 10 minutes at 4°C. Supernatants were collected and protein concentration was determined using the BCA protein assay kit. Proteins were separated by SDS-PAGE and transferred to PDVF membranes. After blocking, the membranes were incubated with specific antibodies and detected with an enhanced chemiluminescence kit. All antibodies used for Western blotting are listed in the supplemental table 3.

Enzyme-linked immunosorbent assay (ELISA)

After co-culturing PBMC with ovarian cancer cells, supernatants were obtained after centrifuged at 1000 g for 5 min. And then assessed IL-6, IL-10, TNF-α, TGF-β, M-CSF, GM-CSF and CCL18 levels by Enzyme-linked immunosorbent assay with specific kits according to the instructions. The O.D. values were measured at 450 nm

on a microplate reader (SynergyH1, BioTek, USA).

Statistical analysis

Quantitative data are mean ± SD from at least 3 independent experiments. Statistical analyses were carried out using SPSS 26.0 software and performed with Grphpad Prism 6 software. The differences in results of Western blot, qRT-PCR, cell-proliferation assays, immunostainings, FCM, and tumor growth were analyzed by Student's t test. $P<0.05$ was considered statistically significant.

Supplemental table 1. The information of low malignant patients and high malignant patients.

sample	Metastasis	Ki67	p53	Vimentin	No. of OvCa-TAMs spheroids	malignancy
1	Abdominal cavity	5%	7%	-	0	Low
2	Abdominal cavity	7%	8%	-	1	Low
3	Abdominal cavity	15%	10%	-	2	Low
4	Abdominal cavity and Lung	50%	63%	+	3	High
5	Abdominal cavity and Bone	75%	80%	+	5	High
6	Abdominal cavity, Lung and Bone	90%	90%	+	6	High

Supplemental table 2. Primes for qRT-PCR

	Forward Sequence	Reverse Sequence
E-CAD	AGGATGACACCCGGGACAAC	TGCAGCTGGCTCAAGTCAAAG
ZEB1	GAAAGTGATCCAGCCAAATGGAA	TTTGGGCGGTGTAGAATCAGAG
SNAIL	CCAGACCCACTCAGATGTCAAG	GGGCAGGTATGGAGAGGAAGA
TWIST1	CAGCTACGCCTTCTCGGTCT	CTGTCCATTTTCTCCTTCTCTGG
M-CSF	GATGGAGACCTCGTGCCAAATTA	TGTTATCTCTGAAGCGCATGGTG
GM-CSF	CATGATGGCCAGCCACTACAA	ACTGGCTCCCAGCAGTCAAAG
β-Actin	TGGCACCCAGCACAATGAA	CTAAGTCATAGTCCGCCTAGAAGCA
EGF	TGGATGTGCTTGATAAGCGG	ACCATGTCCTTTCCAGTGTGT

Supplemental table 3. List of antibodies in Western blot

Antibody name	Company	Host	Cat No.	Dilution
EGF	abcam	rabbit	ab9695	1:1000
E-CAD	CST	mouse	#14472	1:1000
ZEB-1	Abcam	mouse	ab181451	1:500
SNAIL	CST	mouse	#3895	1:1000
TWIST	Abcam	mouse	ab50887	1:500
CCR8	Abcam	rabbit	ab32131	1:500
β-Actin	CST	rabbit	#4970	1:2000

Supplemental figures (SI)

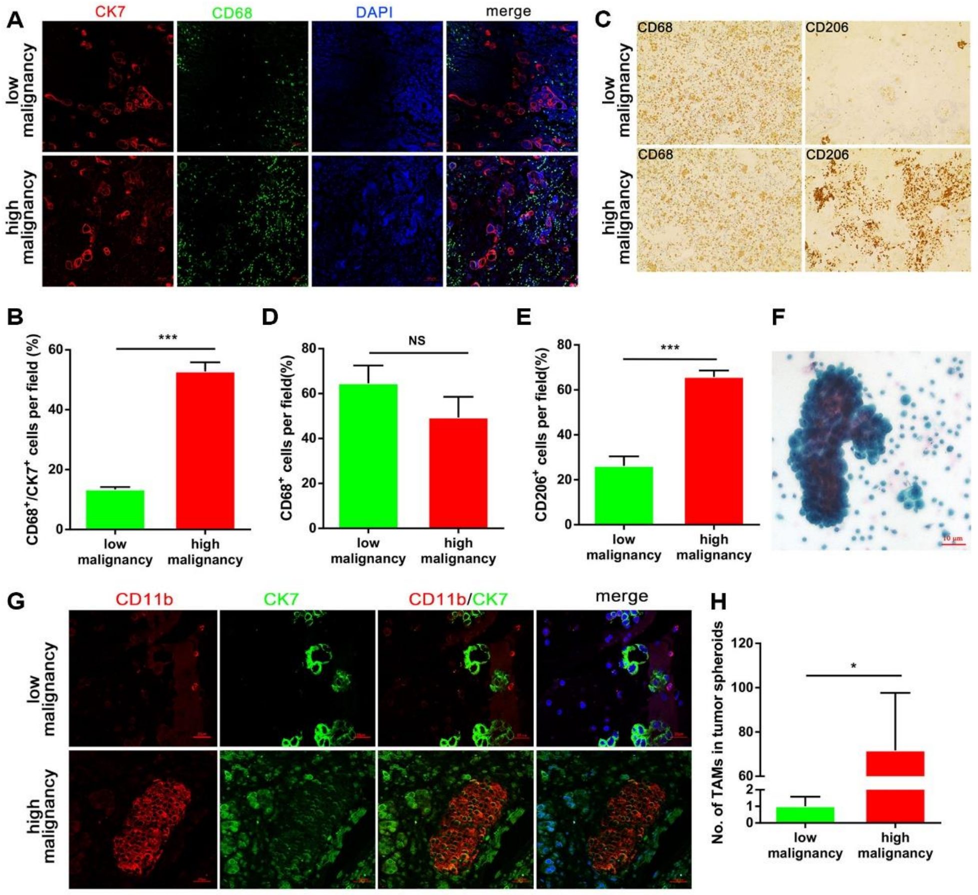

SI. 1. Macrophages are involved in the progression of ovarian cancer. A. OvCa cells and macrophage at the front of the solid tumor were stained and observed under a fluorescence microscope. n = 20 patients. B. the ratio of CD68$^+$ cells and CK7$^+$ cells was quantified. C. Peritoneal cells were smeared on slides and were stained by immunohistochemistry (IHC). D. The average percentage of CD68$^+$ cells per field was quantified. E. The average percentage of CD206$^+$ cells per field was quantified. F. Ascite were smeared on the slides and spheroids were observed. G. Spheroids were subjected to immunostaining with anti-CD11b and anti-CK7, followed by confocal imaging. CD11b$^+$ macrophages, CK7$^+$ tumor cells, and DAPI for nuclear staining are

shown. A merged image is shown on the right. H. Number of TAMs in spheroids was quantified. All data are presented as mean ± SD. n = 20. *P < 0.05; **P < 0.01; ***P < 0.001 (2-sided Student's t test).

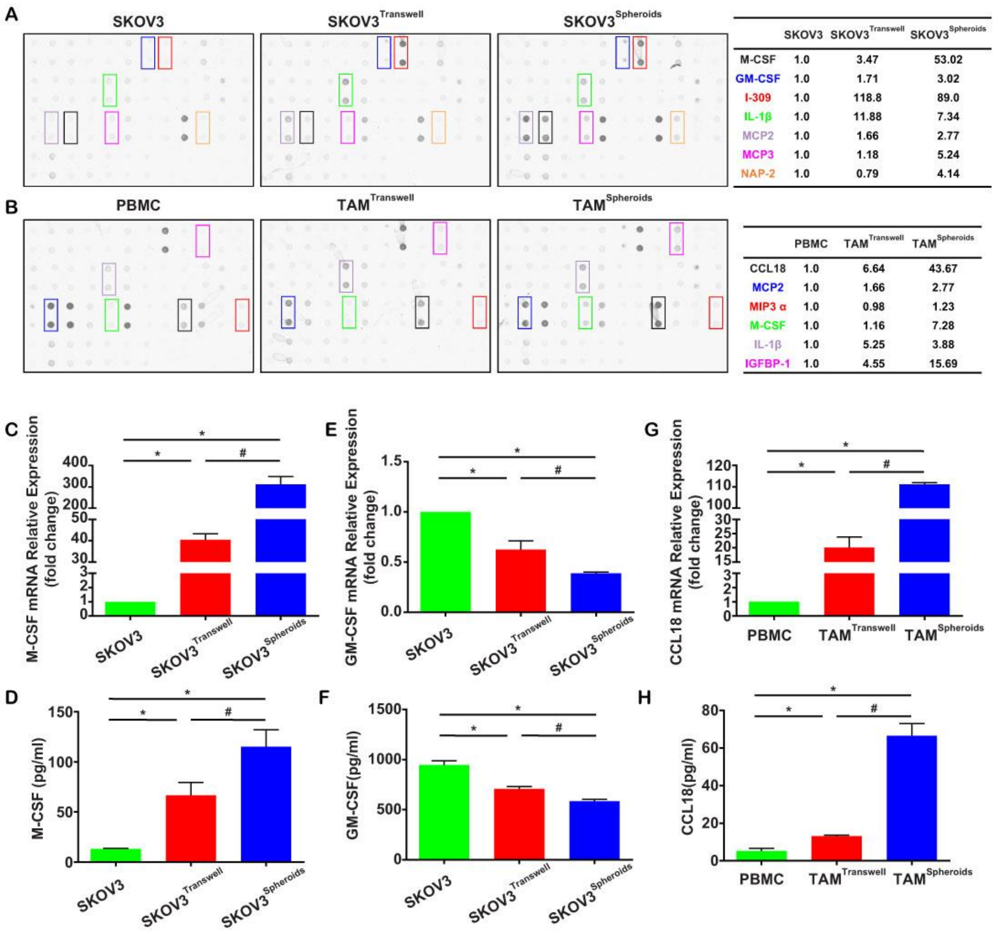

SI. 2. SKOV3 and macrophage in spheroids secrete increase level of M-CSF and CCL18 respectively. A. Cytokine array of the CM of SKOV3, SKOV3^{transwell}, and SKOV3^{spheroids}. The quantified signal intensity of different expression cytokines is showed in the right corner. B. Cytokine array of the CM of PBMC, PBMC^{transwell}, and PBMC^{spheroids}. The quantified signal intensity of different expression cytokines is showed in the right corner. C-F. qRT-PCR analysis

and ELISA analysis of M-CSF and GM-CSF in SKOV3, SKOV3^{transwell}, and SKOV3^{spheroids}. G & H. qRT-PCR analysis and ELISA analysis of CCL18 in PBMC, PBMC^{transwell}, and PBMC^{spheroids}. Data are presented as means ± SD. *P < 0.05; **P < 0.01 (two-sided student's t test).

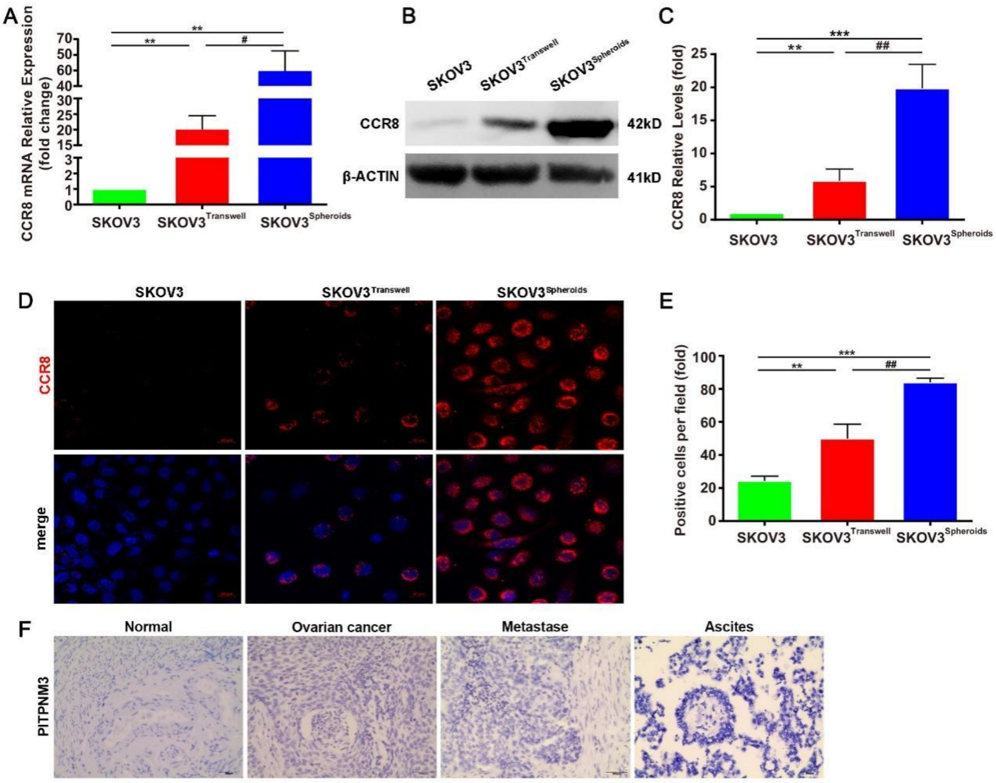

SI. 3. The expression of CCR8 in the OvCa cells. A-C qRT-PCR and Western blot analysis the gene and protein level of CCR8 in the SKOV3, SKOV3^{transwell}, and SKOV3^{spheroids}. Relative protein levels of CCR8 were quantified. D. Immunostaining of CCR8 in the SKOV3, SKOV3, SKOV3^{transwell}, and SKOV3^{spheroids}. CCR8^{+} cells in all groups were quantified in the left corner. The data are presented as means ± SD, n = 5 independent experiments, significant difference are indicated (* p < 0.05, *** p < 0.001 against control). F. The expression of PITPNM3 in the normal ovarian tissue, ovarian cancer tissue, ovarian metastatic colonization and ascites.

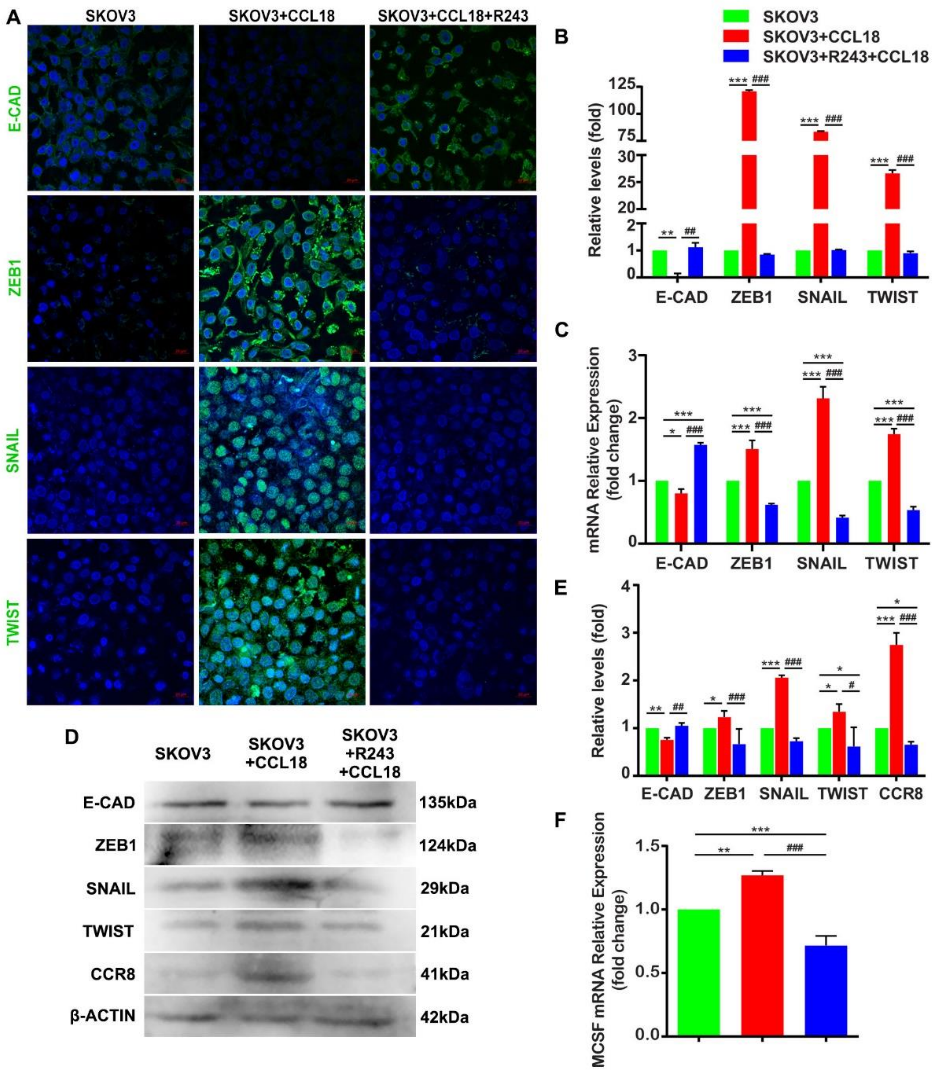

SI. 4. CCL18 induced the EMT of OvCa cells through CCR8. A. Immunofluorescence analysis of E-CAD, ZEB-1, SNAIL and TWIST in SKOV3 cells alone or treated with CCL18, or treated with R243 and CCL18. B. Relative number of E-CAD⁺ SKOV3，ZEB-1⁺ SKOV3, SNAIL⁺ SKOV3 and TWIST⁺ SKOV3 were quantified. C & D. qRT-PCR and Western bot analysis the gene and protein expression of EMT markers in SKOV3 cells alone or treated with CCL18, or treated with R243 and CCL18. E. Relative protein levels of EMT markers were quantified. F.

qRT-PCR analysis the gene expression of M-CSF. The data are presented as means ± SD, n = 5 independent experiments, significant difference are indicated (* $p < 0.05$, *** $p < 0.001$ against control)

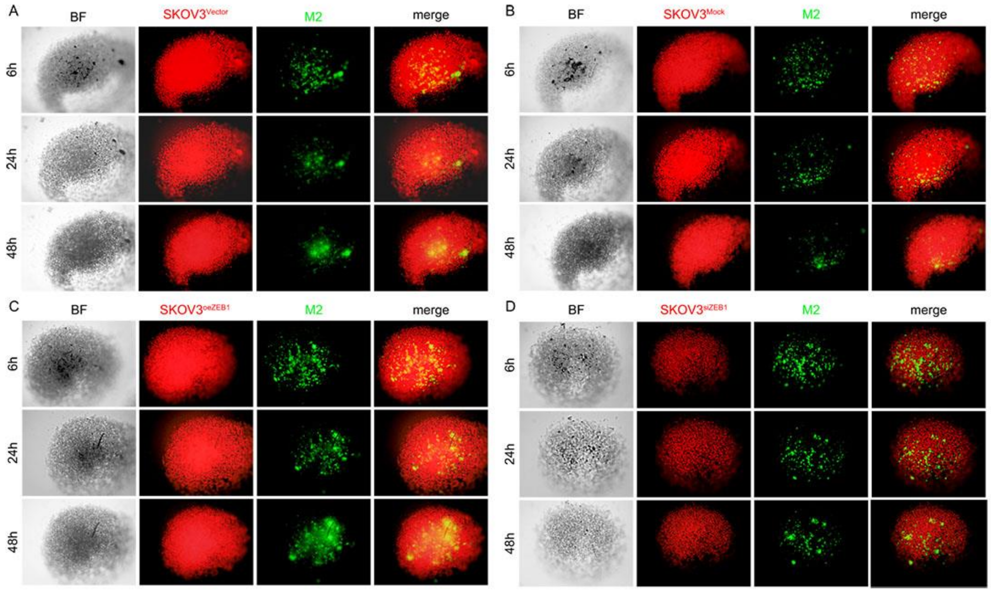

SI. 5. The formation of SKOV3-TAMs spheroids, SKOV3^{oeZEB1}-TAMs spheroids and SKOV3^{siZEB1}-TAMs spheroids 6h, 24h, and 48h co-culture in the ultra-low-attachment 96-well plates 3D co-culture system.

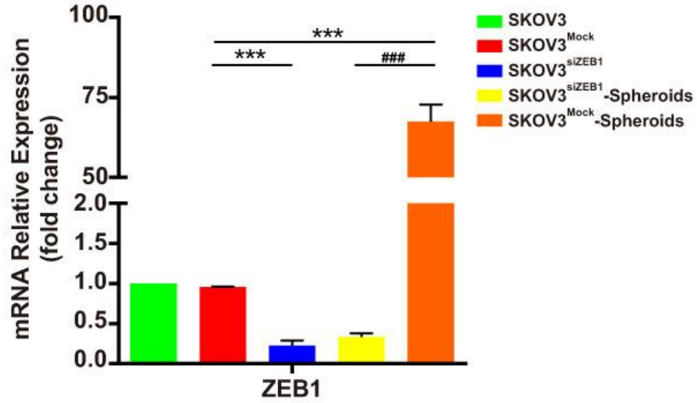

SI.6. The ZEB1 expression level in SKOV3 and in SKOV3 from spheroids.

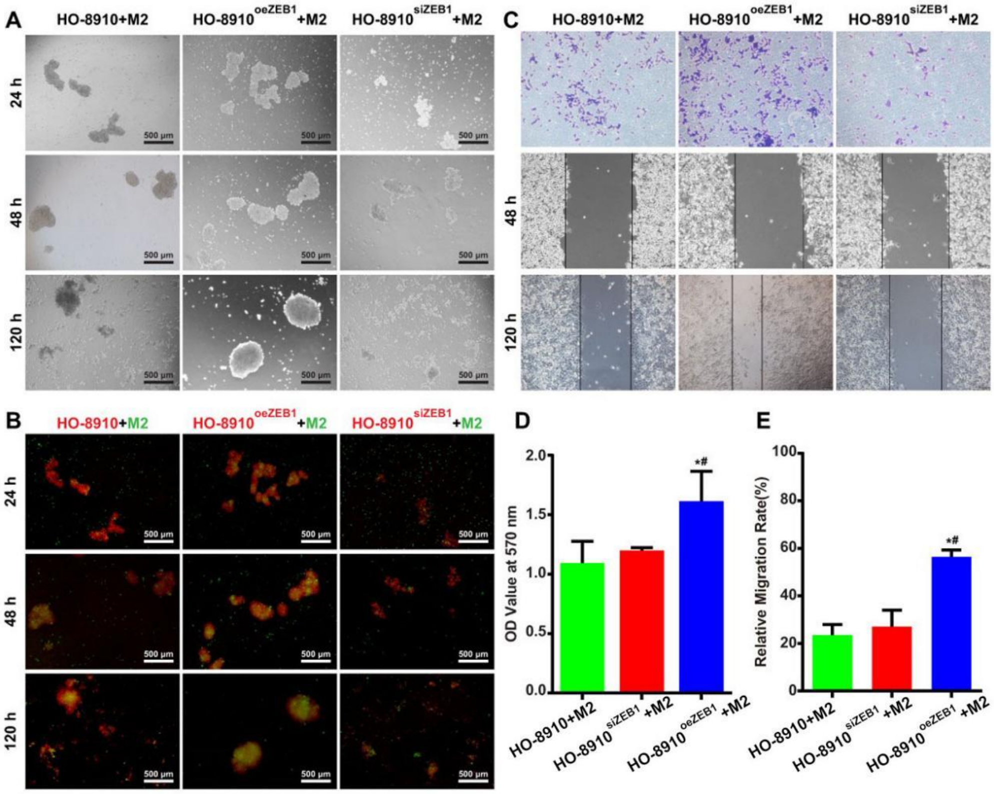

SI. 7. ZEB1 regulate the formation of HO8910-TAMs spheroids. A. The formation of HO8910-TAMs spheroids, HO8910^{oeZEB1}-TAMs spheroids and HO8910^{siZEB1}-TAMs spheroids in the 3D co-culture system. B. The invasive and migratory capacity of HO8910 isolated from

HO8910-TAMs spheroids, HO8910^{oeZEB1}-TAMs spheroids and HO8910^{siZEB1}-TAMs spheroids were measured by transwell-migration assay and scratch wound healing assay. C. The quantitation of transwell-migration assay for isolated HO8910 (n = 4 biologically independent samples per group and an average of five fields acquired from each sample). D. The quantitation of scratch wound healing assay for is olated HO8910 (n = 4 biologically independent samples per group and an average of five fields acquired from each sample).

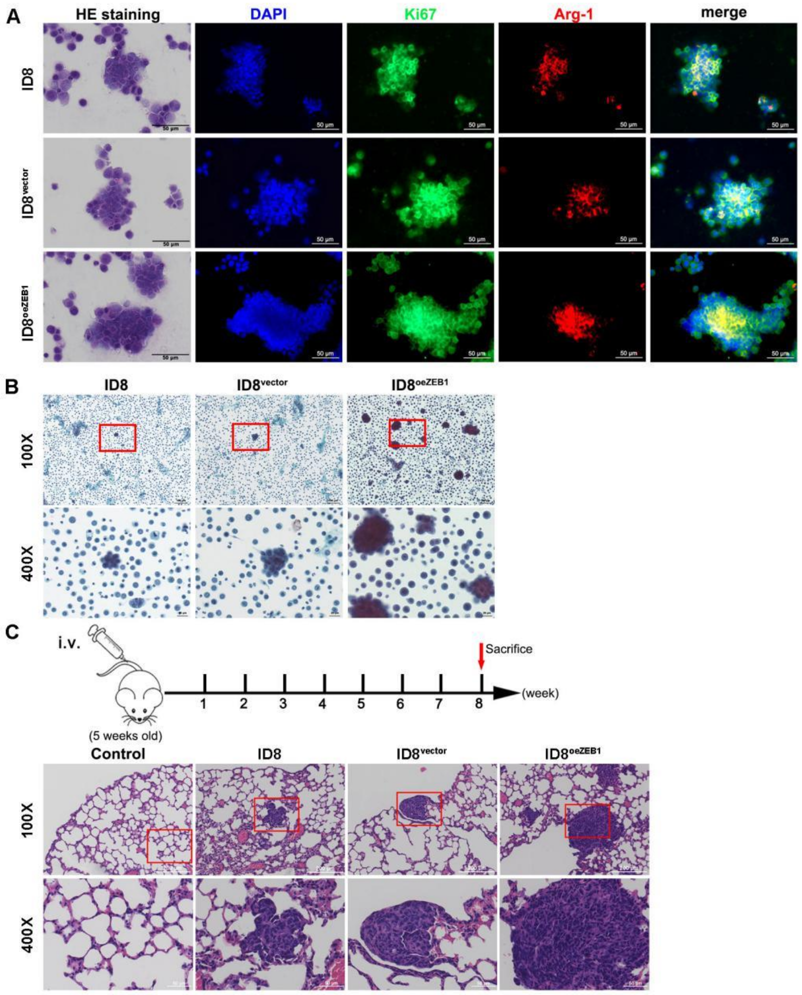

SI. 8. ZEB1 regulate the metastatic process in the OvCa mice model. A & B. The spheroids in the ascites of OvCa mice model constructing by intraperitoneal injection of ID8, ID8^{vector} and ID8^{oeZEB1}. C. Schematic depiction of our in vivo experimental design. The OvCa metastatic mice model constructing by tail intravenous injection of of ID8, ID8^{vector} and ID8^{oeZEB1}. At the 8th week after injection, the mice were sacrificed and the lung tissue was used to perform H&E staining.
